# Genome-Wide Identification and Expression Profiling of the TCP Family Genes in Spike and Grain Development of Wheat (*Triticum aestivum* L.)

**DOI:** 10.3389/fpls.2018.01282

**Published:** 2018-09-10

**Authors:** Junmin Zhao, Zhiwen Zhai, Yanan Li, Shuaifeng Geng, Gaoyuan Song, Jiantao Guan, Meiling Jia, Fang Wang, Guoliang Sun, Nan Feng, Xingchen Kong, Liang Chen, Long Mao, Aili Li

**Affiliations:** National Key Facility for Crop Gene Resources and Genetic Improvement, Institute of Crop Science, Chinese Academy of Agricultural Sciences, Beijing, China

**Keywords:** wheat, TCP, spike, grain, gene family

## Abstract

The TCP family genes are plant-specific transcription factors and play important roles in plant development. TCPs have been evolutionarily and functionally studied in several plants. Although common wheat (*Triticum aestivum* L.) is a major staple crop worldwide, no systematic analysis of TCPs in this important crop has been conducted. Here, we performed a genome-wide survey in wheat and found 66 TCP genes that belonged to 22 homoeologous groups. We then mapped these genes on wheat chromosomes and found that several TCP genes were duplicated in wheat including the ortholog of the maize *TEOSINTE BRANCHED 1*. Expression study using both RT-PCR and *in situ* hybridization assay showed that most wheat TCP genes were expressed throughout development of young spike and immature seed. *Cis*-acting element survey along promoter regions suggests that subfunctionalization may have occurred for homoeologous genes. Moreover, protein–protein interaction experiments of three TCP proteins showed that they can form either homodimers or heterodimers. Finally, we characterized two *TaTCP9* mutants from tetraploid wheat. Each of these two mutant lines contained a premature stop codon in the A subgenome homoeolog that was dominantly expressed over the B subgenome homoeolog. We observed that mutation caused increased spike and grain lengths. Together, our analysis of the wheat TCP gene family provides a start point for further functional study of these important transcription factors in wheat.

## Introduction

TCP genes as plant-specific transcription factors (TFs) are widely present in plants. Previous study showed that TCP genes are involved in a number of critical biological processes, including plant growth, development, and stress responses (Palatnik et al., 2003; Ori et al., 2007; Nag et al., 2009; Danisman, 2016). The name of TCP is derived from three important genes, i.e., the maize *TEOSINTE BRANCHED 1* (*TB1*) that is a major determinant of strong apical dominance in domesticated maize (Doebley et al., 1997), the snapdragon *CYCLOIDEA* (*CYC*) which is involved in the control of floral bilateral symmetry (Luo et al., 1996), and the rice *PROLIFERATING CELL FACTORS 1/2* (*PCF1/2*) that regulate *PROLIFERATING CELL NUCLEAR ANTIGEN* (*PCNA*) for DNA replication and repair (Kosugi and Ohashi, 1997). The TCP domain is composed of 59 amino acid residues, forming a basic helix-loop-helix (bHLH) type of DNA-binding domain non-canonical to regular bHLH TFs (Murre et al., 1989; Cubas et al., 1999). Based on its TCP domain, the members of the TCP family can be grouped into two subfamilies: class I (PCF or TCP-P class) and class II (TCP-C class) (Kosugi and Ohashi, 2002; Navaud et al., 2007; Martín-Trillo and Cubas, 2010). The difference between the two is a four-amino-acid deletion in the TCP domain in class I compared with those of class II. The members of class II are quite heterogeneous and can be further divided into two subclades: CIN and CYC/TB1 subclades (Martín-Trillo and Cubas, 2010). Outside the TCP domain, several class II members process an 18–20-residue arginine-rich motif called the R domain with an unknown function (Cubas et al., 1999).

Homology searches in complete genomes identified 31 TCPs in maize (*Zea mays*) and 24 in sorghum (*Sorghum bicolor* L.) and rice (*Oryza sativa*) (Mondragon-Palomino and Trontin, 2011). The maize TCP genes are distributed unevenly on 10 chromosomes. Based on phylogeny, maize TCP genes are categorized into nine subclasses and purifying selection is assumed to be responsible for maintaining their functions (Chai et al., 2017). Further analysis of maize TCP genes show that they express in stem and ears, suggesting their roles in the development of these two organs. On the other hand, a systematic analysis of sorghum TCP genes (SbTCPs) show that, except for *SbTCP8*, all are either intronless or contain introns in the untranslated regions. Seven pairs of paralogous TCP genes are identified from sorghum, five of which seem to predate rice-sorghum divergence, with diverged expression patterns. Five sorghum TCPs are considered to regulate plant morphology, whereas three genes have been identified as candidates for engineering abiotic stress tolerance (Francis et al., 2016).

In rice, a transcriptome profiling revealed differential accumulation of TCP genes during panicle initiation, organ development, and early anther development. Meanwhile, rice TCP genes are also differentially expressed in response to abiotic stress treatments such as cold, drought, and salt (Sharma et al., 2010). The rice ortholog of the maize *TB1* gene is also expressed in axillary buds and appears to play similar functions as a negative regulator for lateral branching (Takeda et al., 2003). Additional functions were found for other rice TCP genes. The rice *REP1* (*RETARDED PALEA1*) encodes a homolog of the CYC-type gene and during early flower development only expresses in palea primordium. At later floral development stages, *REP1* is radially dispersed in stamens, the vascular bundles of lemma, and palea (Yuan et al., 2009). Another rice TCP gene, *OsTCP19*, appears to be an important node in cell signaling which crosslinks stress and developmental pathways (Mukhopadhyay and Tyagi, 2015). *OsPCF2* may activate *OsNHX1* gene expression, which responds to salt and PEG-induced drought stress, while *OsPCF2* may be associated with the salt and drought stress tolerance (Almeida et al., 2017). Like in other species, some rice TCP genes are targets of microRNA319 (miR319). Transgenic rice plants overexpressing miR319 or down regulating *TCP21* exhibited disease-like phenotypes and showed significantly higher susceptibility to RRSV virus in comparison with the wild-type plants. In rice, the induction of miR319 by RRSV infection suppresses Jasmonic acid (JA)-mediated defense and facilitates virus infection and symptom development by down regulating *TCP21* expression level (Zhang et al., 2016).

Common wheat (*Triticum aestivum* L.) is a staple crop worldwide. Recent availability of wheat genomes allows detailed analysis of gene families in the wheat genome^1^. The wheat *TB1* ortholog, *TaTB1*, has been confirmed to be involved in regulating wheat plant architecture (Liu et al., 2017) and to coordinate axillary spikelet formation during the vegetative to floral transition (Dixon et al., 2018). Except for *TaTB1*, no further analysis of TCP genes and the gene family analysis have been conducted so far. Here, we performed a comprehensive study of TCP genes in wheat. A total of 66 TCP genes belonging to 22 homoeologous groups were found in common wheat and were characterized in detail, including gene/protein architectures, domain conservation, physical properties, chromosomal location, phylogenetic relationship, and *cis*-elements of promoters. We also studied their tissue-specific expression patterns at the wheat heading date, their patterns of expression during young spike and immature seed development, and protein–protein interaction capabilities of some TCP proteins. Finally, two *TaTCP9* mutants from durum wheat were characterized for its functions in spike and grain lengths. Together, our data provide valuable information for further investigation of the molecular functions of TCP genes in wheat which may be useful for wheat genetic improvement.

## Materials and Methods

### Identification of TCP Genes in the Wheat Genome

Wheat protein dataset was downloaded from https://urgi.versailles.inra.fr/download/iwgsc/IWGSC_RefSeq_Assemblies/v1.0/ and were searched using *Arabidopsis* and rice TCP protein sequences as queries using the BLASTP program (*p*-value <= 1e-5). After removing the redundant hits, the presence of TCP domains was verified by searching Pfam and SMART^2^, with ambiguous sequences being manually confirmed using the InterProScan program. Finally, the rice TCP genes were followed to name the wheat TCP genes. Biochemical properties, such as the molecular weight (kDa) and isoelectric point (pI) of each protein, were determined using the Compute pI/Mw tool on the ExPASy website^3^.

### Phylogenetic Analysis and Gene Localition

Multiple sequence alignments were generated using Cluster X (v2.0) with default settings (Larkin et al., 2007). An unrooted phylogenetic tree was constructed using MEGA7.0 software using the neighbor joining (NJ) method, with a bootstrap number of 1000. Chromosomal locations of TCP genes were determined by searching the wheat genomic sequences. The software CIRCOS was used to draw the diagram showing TCP locations and homology relationships on wheat chromosomes that was downloaded from http://mapinspect.software.informer.com/.

### Gene Structure Analysis and Identification of Conserved Motifs

The genomic and coding sequences of TCP genes, together with their exon/intron structures, were extracted from the general feature format (GFF3) file of wheat genome sequences. Additional conserved motifs were checked using the online Multiple Expectation Maximization for Motif Elicitation (MEME) program. The repetition was set as any number with an optimal width of 6–200 residues and the maximum number of motifs as 10.

### *Cis*-Acting Element Analysis and miR159/miR319 Target Site Prediction

The putative promoter sequence, 1.5 kb upstream the transcription start site of each *TaTCP* gene (Zhao et al., 2014; Wang and Liu, 2015), was extracted from wheat genome sequences according to the general feature format (GFF3) file. *Cis*-acting elements were predicted at PlantCARE and those for plant growth, plant development, and phytohormone responses were counted. Using the Analysis of Motif Enrichment (AME) function in the MEME program^4^, enrichment analysis was performed to identify regulatory elements within a collection of promoter sequences from all genes. A set of randomly interrupted promoters were used as a control. The motif with an adjusted Fisher’s test *p*-value less than 0.05 was considered to be a significantly enriched one.

To predict miR159 and miR319 target sites, full-length *TaTCPs* nucleotide sequences were analyzed using the psRNATarget online application. The cutoff of the maximum expectation is four.

### Plant Growth and Tissue Collection

The wheat landrace Chinese Spring (CS) was grown in a farm in Beijing (39.97°N, 116.34°E) for two consecutive springs (2016 and 2017). The developmental stages of the young inflorescences were checked under a stereomicroscope (S8 APO, Leica Microsystems). According to Waddington et al. (1983), spikes at seven developing stages were collected. These stages included W2, W2.5, W3.5, W5.5, W6.5, W7.5, and W8.5. Various tissues including glume, lemma, palea, anther, pistil, and rachis of spike from the middle section of a spike were collected and employed to confirm tissue-specific expression genes at the W9.5 stage. We determined W10 stage as 0 day after pollination (DAP), and collected seeds (4–5 spikelets in the middle section of the spike) for 0, 2, 4, 6, 8, and 10 DAP. Three biological replicates were collected for all samples which were hand-dissected and immediately submerged in liquid nitrogen. Then they were stored at -80°C before RNA extraction. Simultaneously, samples of W3.5, W5.5, and W6.5 were prepared for *in situ* hybridization assays. Seeds for *in situ* hybridization were collected from CS plants that were grown in a glass house with day/night temperatures as 22°C/20°C and light illumination of 16 h/8 h light/dark. The relative humidity was 50%.

### Mutant Identification

Two EMS mutant lines L2431 and L3090 derived from Kronos (Uauy et al., 2009), a desert durum wheat cultivar, were obtained from Drs. Daolin Fu and Jiajie Wu of Shandong Agriculture University, Shandong, China that were originally generated by Dr. Jorge Dubcovsky at UC Davis after Blast searches using wheat TCP sequences (Krasileva et al., 2017). DNA was extracted using the CTAB method as described elsewhere. DNA of mutation sites were PCR amplified and confirmed before further analysis of the mutants.

### RNA Isolation and Real Time Quantitative RT-PCR Analysis

Total seed RNA was extracted using RNAprep Pure Plant Kit (Polysaccharides and Polyphenolics-rich, TianGend) and other total RNA was prepared using TRIzol reagent (Invitrogen). Quantitative real time (RT) PCR was carried out using an ABI PRISM 7300 RT-PCR system. The thermal cycling conditions were one cycle at 95°C for 5 min and 40 cycles of 95°C for 10 s, 55°C for 10 s, and 72°C for 30 s. The relative mRNA level of a gene was calculated as 2^-DDCT^ value using the wheat *GAPDH* (glyceraldehyde-3-phosphate dehydrogenase) gene as an internal control for normalization. Each cDNA sample was tested with three replications. The primer 5 software used for primer design from conserved regions between A, B, and D subgenome with amplified fragments 200–350 bp (**Supplementary Table S1**).

### Yeast Two-Hybrid Assay

The coding sequences of wheat TCP genes were cloned into the bait vector pGBKT7 and/or the prey vector pGADT7 at different restriction sites for yeast two-hybrid assay. The two vectors were then co-transformed into Y2HGold yeast strain (Clontech). After being incubated on double dropout (DDO) medium at 30°C for 2–3 days, yeast cells were selected on DDO medium lacking Leu and Trp. Colonies grown up were further selected on quadruple dropouts (QDO) media lacking Leu, Trp, His and Ade, or on triple dropouts (TDO) media lacking Leu, Trp, and His with 1 mM 3-amino-1,2,4-triazole (3-AT) to identify colonies with positive protein–protein interactions. Empty vectors were used as controls.

### *In situ* Hybridization

RNA *in situ* hybridization was performed according to Liu et al. (2013). Young spikes and immature grains were fixed overnight in formalin-acetic acid-alcohol at 4°C. The samples were then dehydrated through a standard series of ethanol solutions and were embedded in Paraplast Plus (Sigma-Aldrich). A microtome (RM2235, Leica Microsystems) was used to cut tissues into 8 mm sections. Gene-specific regions were amplified and used to synthesizing Digoxigenin-labeled sense and antisense RNA probes using a DIG northern Starter Kit (Roche), according to the manufacturer’s instructions. The sequences of primers are listed in **Supplementary Table S1**.

## Results

### Identification, Phylogeny, and Classification of TCP Genes in Wheat

To identify wheat TCP genes, we used rice and *Arabidopsis* TCP protein sequences as queries and searched the wheat protein dataset at IWGSC^5^ using Blastp. A total of 66 TCP genes were retrieved according to their similarity to query TCP genes and the presence of TCP domains and conserved motifs. The serial numbers of wheat TCP genes were named according to their best rice homologs (**Table 1**). All wheat TCP genes had three homoeologs.

**Table 1:** Wheat TCP family genes.

Gene name	Gene ID	ORF length	No. intron	pI	Mw	Chromosome location	Subclade
TaPCF5-A	TraesCS3A01G140100.1	1373	2	6.44	47915.75	chr3A:119137149-119140425	CIN
TaPCF5-B	TraesCS3B01G164500.1	1352	2	6.37	47007.34	chr3B:162222339-162224970	CIN
TaPCF5-D	TraesCS3D01G146900.1	1364	2	6.41	47521.58	chr3D:111206159-111208623	CIN
TaPCF6-A	TraesCS5A01G423900.1	1217	1	9.59	38470.11	chr5A:609619180-609621983	CIN
TaPCF6-B	TraesCS5B01G426200.1	1214	1	9.39	41923.59	chr5B:601864073-601867389	CIN
TaPCF6-D	TraesCS5D01G432500.1	1205	1	9.39	41382.26	chr5D:488677442-488679253	CIN
TaPCF7-A	TraesCS3A01G422500LC.1	1037	intronless	5.62	36063.32	chr3A:524756895-524757932	CIN
TaPCF7-B	TraesCS3B01G503200LC.1	1037	intronless	5.72	36269.33	chr3B:529404878-529405915	CIN
TaPCF7-D	TraesCS3D01G384600LC.1	1043	intronless	5.52	36205.32	chr3D:403111095-403112318	CIN
TaPCF8-A	TraesCS5A01G031900.1	893	2	6.76	32293.85	chr5A:29512544-29516131	CIN
TaPCF8-B	TraesCS5B01G032000.1	902	intronless	6.75	32434.76	chr5B:35188411-35189313	CIN
TaPCF8-D	TraesCS5D01G040100.1	887	2	6.7	31938.64	chr5D:41117072-41120665	CIN
TaTCP5-A	TraesCS3A01G302900.1	836	1	6.95	30140.75	chr3A:536895105-536897646	CIN
TaTCP5-B	TraesCS3B01G333400.1	842	1	7.3	30335.92	chr3B:539942261-539944819	CIN
TaTCP5-D	TraesCS3D01G298900.1	827	1	7.3	29822.66	chr3D:413676020-413678583	CIN
TaTCP18-A	TraesCS1A01G332600.1	920	1	6.72	32919.34	chr1A:520938291-520940709	CIN
TaTCP18-B	TraesCS1B01G346300.1	926	1	7.1	33085.47	chr1B:575440078-575442514	CIN
TaTCP18-D	TraesCS1D01G335300.1	941	intronless	6.41	33678.57	chr1D:425299621-425300562	CIN
TaTCP21-A	TraesCS2A01G251100.1	1646	1	10.02	59214.07	chr2A:379150934-379159267	CIN
TaTCP21-B	TraesCS2B01G265800.1	1274	1	9.18	45596.65	chr2B:357918017-357924451	CIN
TaTCP21-D	TraesCS2D01G252100.1	1250	1	9.42	44631.32	chr2D:302247032-302253739	CIN
TaTB1-1-A	TraesCS4A01G271300.1	1058	intronless	7.15	37911.58	chr4A:582839670-582841244	CYC/TB1
TaTB1-1-B	TraesCS4B01G042700.1	1064	intronless	7.72	38223.75	chr4B:30362277-30363341	CYC/TB1
TaTB1-1-D	TraesCS4D01G040100.1	1058	1	7.71	37835.57	chr4D:18463838-18472387	CYC/TB1
TaTB1-2-A	TraesCS5A01G001900.1	821	intronless	9.46	30056	chr5A:1346522-1347511	CYC/TB1
TaTB1-2-B	TraesCS5B01G002300.1	887	intronless	9.42	32627.27	chr5B:3474782-3475968	CYC/TB1
TaTB1-2-D	TraesCS5D01G002300.1	794	intronless	9.83	29191.66	chr5D:2511000-2512022	CYC/TB1
TaTCP22-A	TraesCS2A01G018000.1	614	intronless	5.42	22126.96	chr2A:8543341-8543955	CYC/TB1
TaTCP22-B	TraesCS2B01G025700.1	614	intronless	5.09	21865.79	chr2B:11836978-11837592	CYC/TB1
TaTCP22-D	TraesCS2D01G019300.1	710	intronless	5.46	25633.9	chr2D:9079476-9080529	CYC/TB1
TaTCP24-A	TraesCS5A01G207300.1	818	intronless	5.82	29889.42	chr5A:419207299-419208117	CYC/TB1
TaTCP24-B	TraesCS5B01G205600.1	824	intronless	5.94	30149.55	chr5B:374190914-374192086	CYC/TB1
TaTCP24-D	TraesCS5D01G213400.1	827	intronless	5.94	30230.53	chr5D:322577220-322578047	CYC/TB1
TaPCF1-A	TraesCSU01G021700LC.1	863	intronless	6.45	13470.32	chrUn:12584619-12585482	PCF
TaPCF1-B	TraesCS2B01G062900.1	482	intronless	9.79	16586.69	chr2B:30145219-30145701	PCF
TaPCF1-D	TraesCS2D01G049400.1	266	intronless	4.56	9326.79	chr2D:18210500-18210766	PCF
TaPCF2-A	TraesCS4A01G355900.1	889	2	5.28	30860.2	chr4A:629486061-629487401	PCF
TaPCF2-B	TraesCS5B01G516200.1	1049	intronless	5.43	36115.83	chr5B:680580273-680583936	PCF
TaPCF2-D	TraesCS5D01G516300.1	1034	1	5.54	35912.84	chr5D:539014262-539015611	PCF
TaPCF3-A	TraesCS4A01G222700.1	1157	intronless	6.98	40576.52	chr4A:530121678-530123374	PCF
TaPCF3-B	TraesCS4B01G093700.1	1145	intronless	6.81	40160.3	chr4B:96270932-96272626	PCF
TaPCF3-D	TraesCS4D01G090500.1	1157	intronless	6.98	40601.52	chr4D:65576568-65578302	PCF
TaTCP6-A	TraesCS3A01G436100.1	1025	1	6.27	35905.49	chr3A:679694378-679696479	PCF
TaTCP6-B	TraesCS3B01G470700.1	1049	2	6.35	36864.9	chr3B:718849770-718851635	PCF
TaTCP6-D	TraesCS3D01G428700.1	1010	2	6.23	35492.25	chr3D:543008179-543010159	PCF
TaTCP7-A	TraesCS6A01G233800.1	611	intronless	9.51	21371.71	chr6A:441486019-441486880	PCF
TaTCP7-B	TraesCS6B01G262600.1	598	intronless	9.51	20937.53	chr6B:473055822-473056442	PCF
TaTCP7-D	TraesCS6D01G216100.1	620	1	9.51	21813.98	chr6D:306129040-306130108	PCF
TaTCP9-A	TraesCS6A01G306500.1	1214	intronless	9.17	40755.88	chr6A:540144123-540146045	PCF
TaTCP9-B	TraesCS6B01G334900.1	1208	intronless	9.19	40408.73	chr6B:589127612-589129644	PCF
TaTCP9-D	TraesCS6D01G285600.1	1205	intronless	9.19	40460.8	chr6D:393934793-393936464	PCF
TaTCP17-A	TraesCS2A01G376000.1	617	intronless	9.61	21774.02	chr2A:618266685-618267625	PCF
TaTCP17-B	TraesCS2B01G392900.1	755	intronless	9.95	26968.79	chr2B:557226759-557227975	PCF
TaTCP17-D	TraesCS2D01G372200.1	617	intronless	9.61	21744.01	chr2D:476344966-476346144	PCF
TaTCP19-A	TraesCS7A01G244300LC.1	680	intronless	9.81	23772.15	chr7A:140536199-140537919	PCF
TaTCP19-B	TraesCS7B01G091900.1	1118	1	9.59	38470.11	chr7B:105322710-105325639	PCF
TaTCP19-D	TraesCS7D01G187600.1	1202	intronless	8.98	41495.36	chr7D:140232150-140234052	PCF
TaTCP25-A	TraesCS5A01G309300.1	1016	Intronless	5.06	34474.11	chr5A:521743536-521744868	PCF
TaTCP25-B	TraesCS5B01G310000.1	1019	intronless	4.95	34568.02	chr5B:491993823-491995127	PCF
TaTCP25-D	TraesCS5D01G316300.1	1004	intronless	5.03	33989.76	chr5D:410240816-410242165	PCF
TaTCP28-A	TraesCS5A01G121800.1	1259	intronless	5.53	43434.48	chr5A:264421099-264422849	PCF
TaTCP28-B	TraesCS5B01G117100.1	1253	intronless	5.66	43278.42	chr5B:206101060-206102857	PCF
TaTCP28-D	TraesCS5D01G199700LC.1	1655	intronless	6.47	57569.72	chr5D:205995024-205997202	PCF
TaTCP29-A	TraesCS6A01G412800.1	596	1	6.34	19973.82	chr6A:614381484-614383233	PCF
TaTCP29-B	TraesCS6B01G462100.1	596	intronless	6.58	20230.98	chr6B:715448937-715450031	PCF
TaTCP29-D	TraesCS6D01G397000.1	593	1	6.54	20051.93	chr6D:469205255-469207520	PCF


*pI, isoelectric point; Mw, molecular weight.*

**FIGURE 1:**
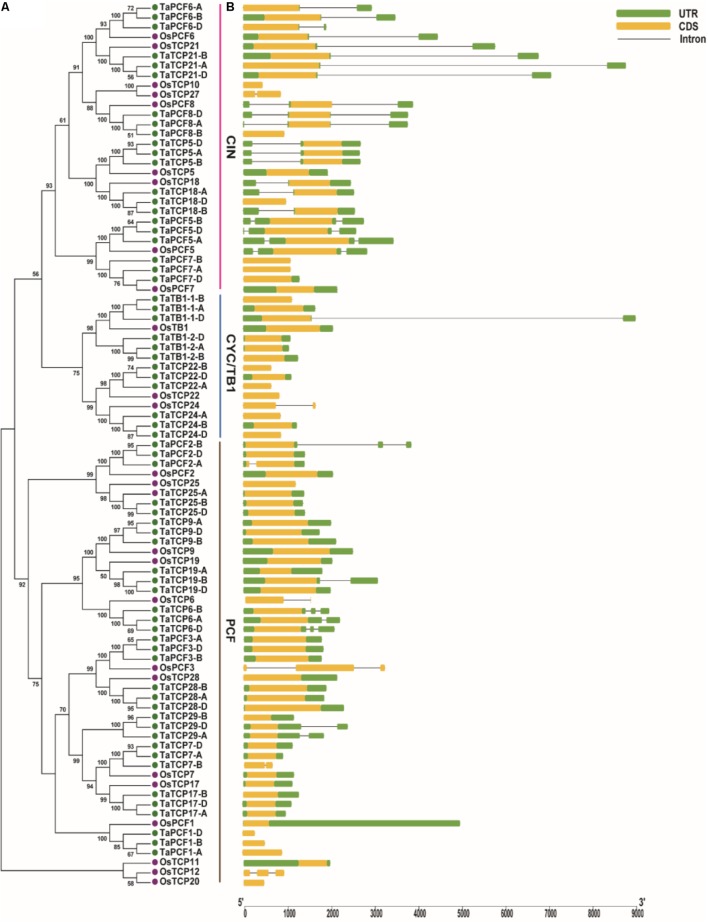
Phylogenetic and gene structure analyses of wheat TCPs. **(A)** A Neighbor Joining tree of wheat TCP proteins. **(B)** Wheat TCP gene structures. UTR, CDS, and intron are represented with green boxes, yellow boxes, and black lines respectively.

**FIGURE 2:**
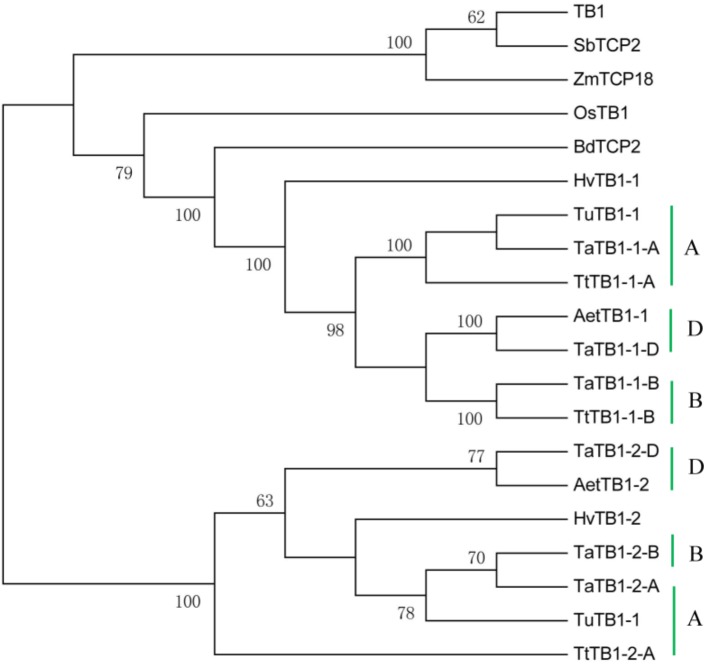
Phylogenetic tree of TB1 orthologs and paralogs from wheat related species *Aegilops tauschii* (Aet, DD), *Triticum urartu* (Tu, AA), tetraploid *Triticum turgidum* (Tt, AABB), barley (Hv), and other monocot species *Brachypodium* (Bd), *Zea mays* (Zm), *Oryza sativa* (Os), and Sorghum (Sb). A, B, and D indicate wheat three subgenomes.

A phylogenetic tree was constructed using neighboring joining (NJ) method so as to distinguish the evolutionary relationship of wheat TCPs. Similar to that in other plants, wheat TCPs can be categorized into two classes: class I (PCF) and class II (CIN and CYC/TB1) (**Figure 1**). Among them, 33 members (TaPCF1, 2, 3; TaTCP6, 7, 9, 17, 19, 25, 28, and 29) were in class I and the remaining fell into class II, including 12 CYC/TB1-type (TaTB1-1, TaTB1-2, TaTCP22, and 24) and 21 CIN-type (TaPCF5, 6, 7, 8; TaTCP5, 18, 21). This is comparable to those in rice where there are 13 class I TCPs and 12 class II TCPs, including three CYC/TB1 type TCPs and nine CIN type TCPs. It is interesting that the wheat TB1 locus was duplicated, one on chromosome 4 and the other on chromosome 5 and each having three homoeologs (**Table 1** and **Figure 1**). The one on chromosome 4 has been shown to regulate inflorescence architecture and development in wheat (Dixon et al., 2018), while there is no functional report on the one on chromosome 5. To confirm this observation, we obtained all *TB1* orthologs and paralogs from wheat donor species including diploid (Aegilops tauschii and Triticum urartu), tetraploid (Triticum turgidum), as well as some other monocots including barley, rice, maize, Brachypodium, and sorghum (**Figure 2** and **Supplementary Table S2**). Phylogenetic analysis showed that wheat related species all had two *TB1* homologs. Maize also had two *TB1* homologs, but seemed to be arising by segmental duplication. In addition, all remaining species contained one *TB1* gene including rice, Brachypodium, and sorghum. Then we calculated the K_s_ and K_a_/K_s_ ratio for each duplicated *TB1* gene pairs (**Table 2**). The results showed that K_a_/K_s_ of all five pairs were <1, indicating the effect of strong purifying selection and the slow evolution rate in TB1 protein sequences in wheat. The duplication of the five paralogous gene pairs was estimated to have occurred 0.62–0.84 million years ago (mya). We also found that there was no wheat ortholog for rice CIN-type TCPs OsTCP10 and 27 and for rice PCF-type TCPs OsTCP11, 12, and 20 (**Figure 1A**). Conversely, wheat TaTCP29 (TaTCP29-A, TaTCP29-B, and TaTCP29-D) had no ortholog in rice. To get a better understanding of the diversification of the TaTCP genes, we analyzed the exon/intron organization of TaTCPs and found that nearly all genes had their introns located at the 5′ or 3′ UTR regions, except for TaPCF2-A and TaTCP7-B which had their introns located in the middle of coding regions (**Figure 1B**).

**Table 2:** K_a_/K_s_ analysis and estimated divergence time for *TaTB1* gene pairs in wheat and its donor species.

Paralogous pairs	Origin	K_a_	K_s_	K_a_/K_s_	Divergence time (mya)
TaTB1-1-A-TaTB1-2-A	AABBDD	0.422331	1.76758	0.238932	0.718717
TaTB1-1-B-TaTB1-3-B	AABBDD	0.39819	1.42517	0.279399	0.61836
TaTB1-1-D-TaTB1-4-D	AABBDD	0.391273	1.89824	0.206124	0.710809
TtTB1-1-A-TtTB1-2-A	AABB	0.389092	1.96989	0.19752	0.71316
AetTB1-1-AetTB1-2	DD	0.366917	2.7072	0.135534	0.839375
TuTB1-1-TuTB1-2	AA	0.366908	1.54729	0.23713	0.615564


### Genomic Organization and Duplication of Wheat TCP Proteins

**FIGURE 3:**
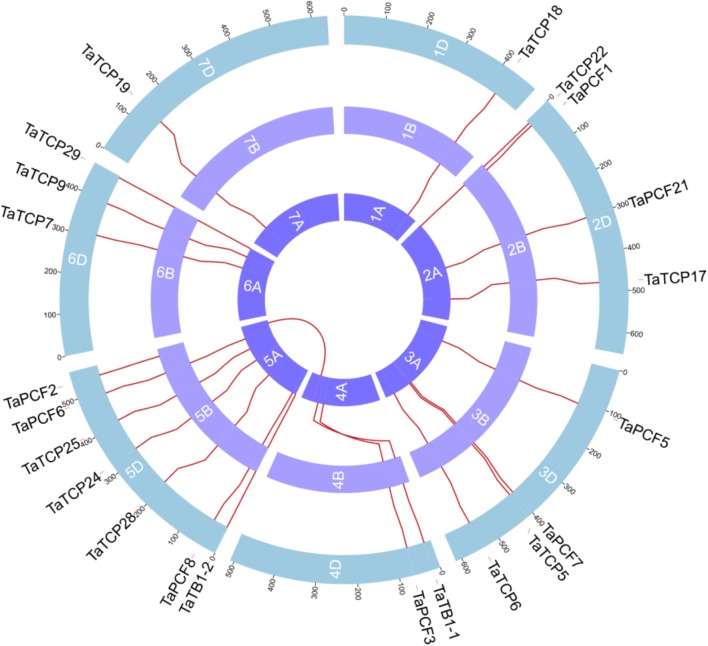
Chromosomal localizations of TCP genes in wheat. TCP genes were mapped on wheat chromosomes by using genomic information available in (https://urgi.versailles.inra.fr/download/iwgsc/IWGSC_RefSeq_Annotations/v1.0/). Red lines represent the synteny of triplets. Different colors represent different linkage groups. Blue, the A subgenome; light purple, the B subgenome; and Purple, the D subgenome.

Wheat TCP genes were found to be located on all chromosomes, with a maximum of seven genes on chromosome 5 and only one on chromosome 1 and 2 (**Figure 3**). The A homoeolog of *TaPCF2* was translocated from 5AL to 4AL which was consistent to previous study (Devos et al., 1995). The A homoeolog of *TaTB1-1* and *TaPCF3* were translocated from 4AS to 4AL, which was confirmed by check locations 100 Mb up and downstream regions of these two genes. Our pipeline also detected the 7BS and 4AL translocation that has been reported previously (Berkman et al., 2012). Interestingly, as mentioned earlier, *TaTB1* was found on two loci, one on chromosome 4 and the other on chromosome 5. This is in contrast with those in rice and maize where only one *TB1* gene was reported (Takeda et al., 2003; Clark et al., 2006).

### Conserved Motifs and Domains on TaTCP Proteins

To identify conserved motif and evaluate structural divergence, TaTCP proteins were further analyzed at the MEME web server. A total of 10 conserved motifs (motif 1–10) were identified (**Supplementary Figure S1**). All but five (TaPCF1-A, D; TaTCP29-A, B, D) had conserved motif 1 and motif 2 that constituted the typical TCP domain. Similar to those reported by Chai et al. (2017), TCP proteins in the same subgroup share similar conserved motifs that were clearly distinguishable from those in other subgroups. CIN-type TCPs formed three subgroups: subgroup 1 had eight motifs (1, 2, 3, 4, 5, 6, 7, and 8); subgroup 2 had four motifs (1, 2, 4, and 8); and subgroup 3 had eight motifs (1, 2, 4, 5, 8, 9, and 10) (**Figure 1** and **Supplementary Figure S1**). CYC/TB1-type TCPs had two subgroups: subgroup 1 conferred five motifs (1, 2, 3, 4, and 8) and subgroup 2 had four motifs (1, 2, 8, and 9), while PCF-type had five subgroups with subgroup 1 having three motifs (1, 2, and 3), subgroup 2 having seven motifs (1, 2, 3, 5, 6, 9, and 10), subgroup 3 having six motifs (1, 2, 3, 5, 6, and 8), subgroup 4 having seven motifs (1, 2, 3, 4, 5, 6, and 8), and subgroup 5 having seven motifs (1, 2, 3, 5, 7, 8, and 9).

**FIGURE 4:**
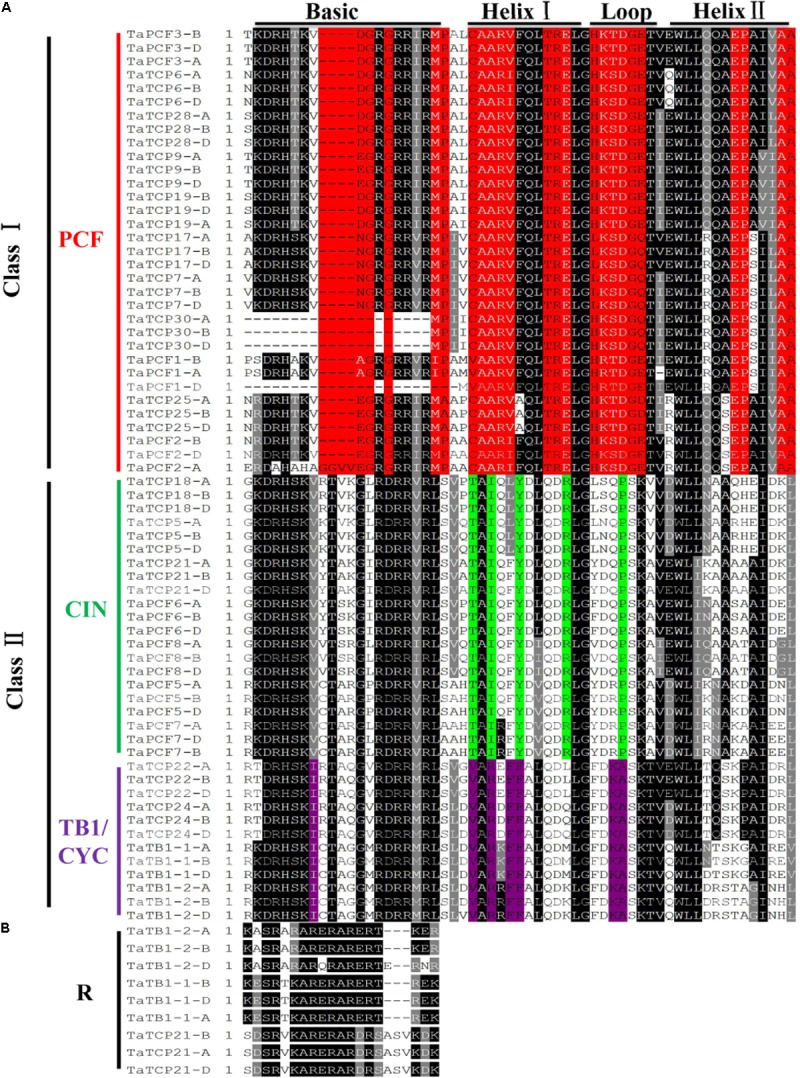
Multiple sequence alignment of wheat TCP transcription factors. **(A)** Alignment of the TCP domain of wheat TCP proteins. Black boxes highlight residues conserved in all three TCP classes; Red, residues conserved in the PCF class; Green, residues conserved in the CIN class; and Purple, residues conserved in the CYC/TB1 class. Domains of the Basic, Helix I, Loop, and Helix II regions are underlined on the top of the alignment. **(B)** Alignment of the R domains.

To identify conserved domains of TCP proteins and to further classify TCP proteins, we searched the TCP domain using Scanprosite and found a total of four conserved motifs – Basic, Helix I, Loop, and Helix II (**Figure 4**). In most cases, TCP proteins in the same subclade shared similar motif compositions. A four-amino-acid deletion was found to distinguish the class I TCPs from those of class II in their basic domain (**Figure 4A**).

A wheat TCP phylogenetic tree was built using full length protein sequences. The structure was similar to those of *Arabidopsis* and rice where all the proteins can be classified into two major classes, with those having a four-amino-acid deletion in the basic region of the TCP domain as class I TCP and those with TCP domains as class II which were further divided into CIN- and CYC/TB1-subclades (Yao et al., 2007). The wheat CYC/TB1 subclade contained four TaTCP genes (*TaTB1-1*, *TaTB1-2*, *TaTCP22*, and *TaTCP24*), and the CIN subclade contained seven (*TaPCF5*, *TaPCF6*, *TaPCF7*, *TaPCF8*, *TaTCP5*, *TaTCP18*, and *TaTCP21*; **Figure 4A**). The R domain was found in a subset of class II proteins (TaTB1-1, TaTB1-2, and TaTCP21) (**Figure 4B**).

### Wheat TCP Genes With microRNA Target Sites

**FIGURE 5:**
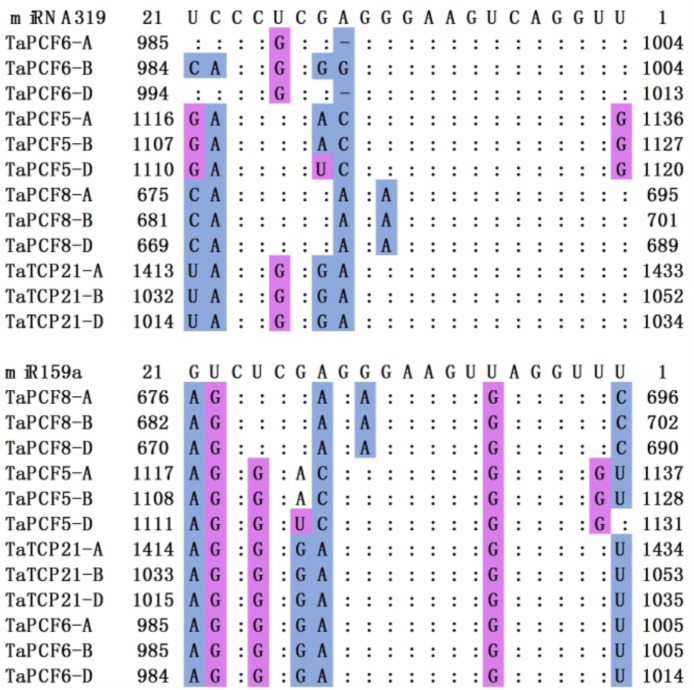
Alignment of putative target areas for miR319 and miR159. Mismatches and G-U wobbles were represented by blue and purple, respectively.

In *Arabidopsis*, CIN-TCP genes are post-transcriptionally regulated by miR319a (Palatnik et al., 2003). In case of the CIN-type TCPs, increased miR319a activity (e.g., jaw-D mutants and miR319a-overexpressing lines) causes simultaneous down regulation of five TCP genes (Nicolas and Cubas, 2016). We found in wheat that *TaPCF5*, *6*, *8*, and *TaTCP21* contained sequences well matched with miR319a and might be the targets of microRNAs (**Figure 5**). This is consistent with those in rice where five genes *OsPCF5*, *6*, *8*, *OsTCP20*, *21*, and *27* are targeted by miR319. Moreover, four wheat TCPs: *TaPCF5*, *6*, *8*, and *TaTCP21*, may be the targets of the second microRNA tae-miR159a (**Figure 5**).

### Identification of *Cis*-Acting Elements in the Promoter of TaTCP Genes

**FIGURE 6:**
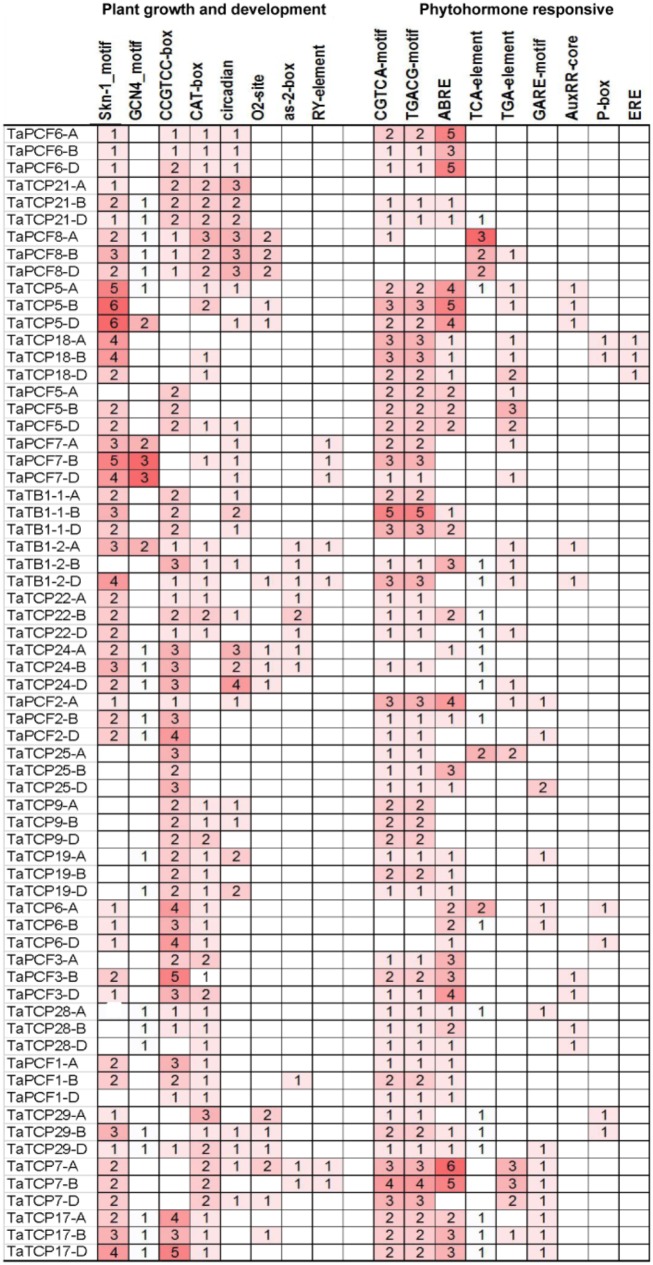
*Cis*-acting elements on promoters of wheat TCP genes.

The distribution of different *cis*-acting elements in the promoter of a gene may indicate the difference in its function and regulation. We extracted 1.5 kb of genomic sequence upstream the translation start site of each TCP gene and searched for *cis*-acting elements in the PlantCARE database (Lescot et al., 2002). *Cis*-elements responsible for plant growth and development and phytohormone responses were identified (**Figure 6**). Two motifs, Skn-1 and GCN4, are involved in endosperm expression (Wang et al., 1999). Skn-1-motif was found in most TaTCP genes (at least in one homoeolog) except for four PCF-type genes (*TaTCP9*, *19*, *25*, and *28*). The meristematic expression and specific activation elements CAT-box and CCGTCC-box were also found in most TaTCP genes. The PCF-type gene *TaTCP7* and the CIN-type genes *TaTCP5*, *TaTCP18*, and *TaPCF7* contained Skn-1 motifs, but no CCGTCC-box element was found in any of its homoeolog. All three homoeologs of CIN-type genes (*TaTCP21*, *TaPCF6*, *7*, and *8*), the PCF-type *TaTCP29*, and the CYC-type *TaTB1-1* and *TaTCP24* were found to bear circadian control element (Anderson et al., 1994). Wheat TCP gene promoters also possessed other *cis*-elements, such as those specific to seed and shoot development (e.g., RY and as-2-box) (Bobb et al., 1997) and those for zein metabolism regulation element (e.g., O_2_ site).

For hormone-related *cis*-acting elements, the MeJA-responsive elements CGTCA and TGACG (Rouster et al., 1997) were most frequently identified at the wheat TCP gene promoters. The ABA-responsive element (ABRE; Shen and Ho, 1995) was also found in most of TaTCP genes. For *TaTCP7*, six and five *cis*-elements were identified on its A and B homoeolog promoters, respectively, but none on that of the D homoeolog. A number of other hormone related *cis*-elements, such as gibberellin responsive elements GARE and P-box and auxin-responsive elements TGA, AUXRR core, and TGA box (Kim et al., 1992; Ulmasov et al., 1997; Washida et al., 1999) were also present in promoters of some wheat TCP genes.

Since *cis*-acting elements are usually very short and not very well defined, to avoid the likelihood of detecting them by chance, we performed *cis*-acting element enrich analysis and confirmed that *cis*-elements responsible for plant growth and development were indeed enriched (**Table 3**).

**Table 3:** Enriched *cis*-acting elements in TaTCP gene promoter regions.

Motif altmetric ID	Consensus	Motif name	*p*-value	Adjusted *p*-value
**Plant development**				
CCGTCC	CCGTCC	CCGTCC-box	4.12E-10	1.52E-07
GATAATGATG	GATAATGATG	as-2-box	1.14E-04	8.61E-03
CATGCATG	CATGCATG	RY-element	1.12E-06	3.34E-04
**Hormone response**				
AAATGGAGA	AAATGGAGA	P-box	9.38E-04	0.204^∗^
AACGAC	AACGAC	TGA-element	8.02E-04	0.274^∗^
**Biotic and abiotic response**				
AAACAGA	AAACAGA	ARE	2.80E-06	5.24E-04
AAAAAATTTC	AAAAAATTTC	HSE	9.13E-08	1.57E-05


*^∗^represents not significant.*

### Spatial and Temporal Expression Patterns of Wheat TCP Genes

**FIGURE 7:**
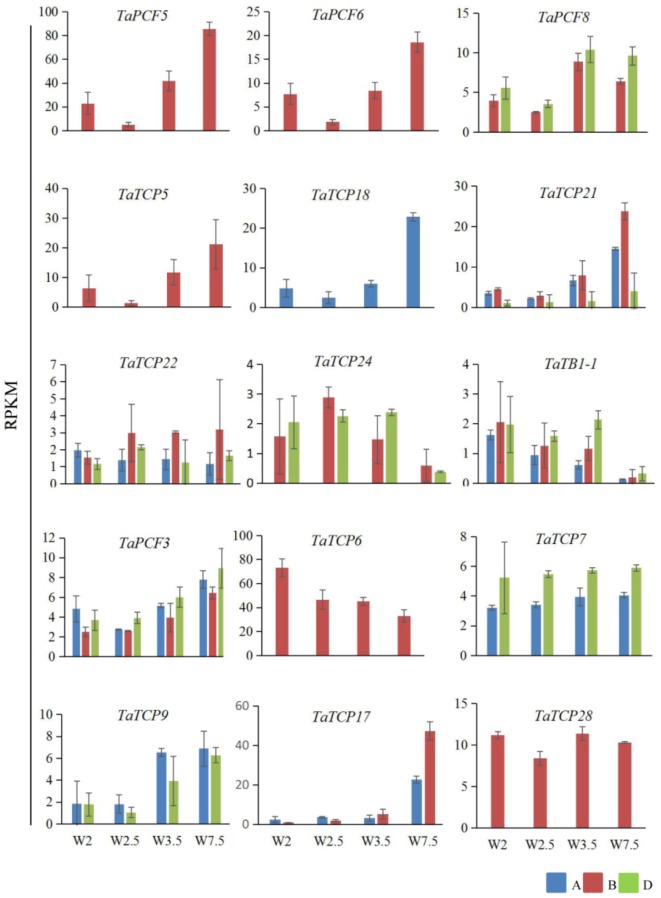
Expression patterns wheat TCP genes during young spike development by RNA-seq. Developmental stages are indicated using Waddington (W) scales (Waddington et al., 1983). W2, double ridge (DR); W2.5, floral meristem stage (FM), W3.5, anther meristem stage (AM); and W7.5, tetrad stage (TS). Y axis is the RPKM (Reads per kilobase per million mapped reads) value; gene names are shown in **Supplementary Table S2**. Each point of the RPKM represents the average of two biological replicates. Error bars indicate SD.

**FIGURE 8:**
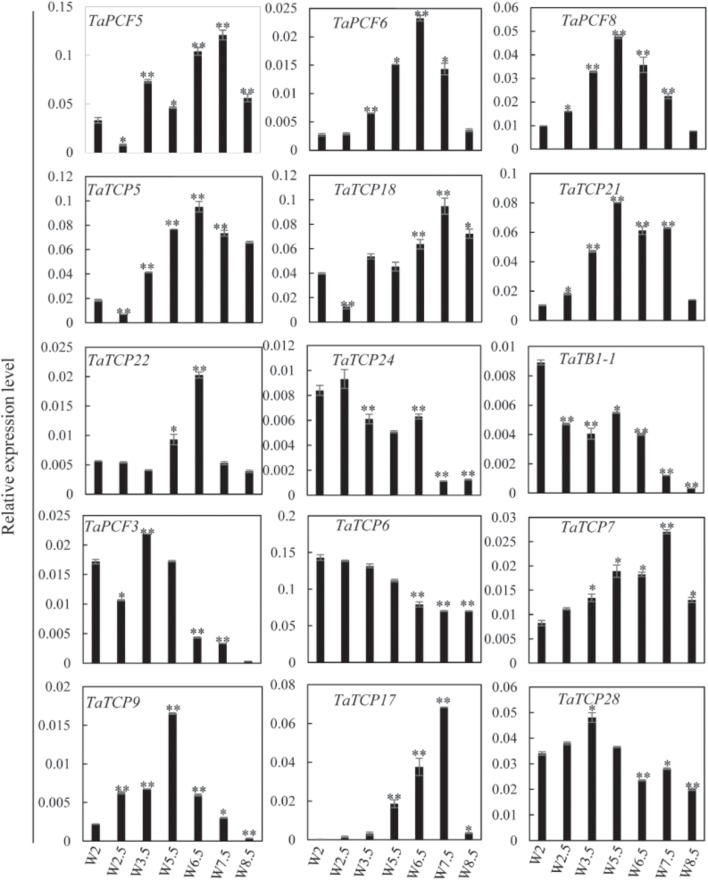
TaTCP gene expression patterns during young spike development by RT-PCR. Developing young spike stages were determined using Waddington (W) scales (Waddington et al., 1983). Three biological duplicates were performed for each gene; Y axis represents the relative expression value to *GAPDH* gene, the internal control. Asterisks indicate significant difference relative to W2, as determined by Student’s *t*-test (^∗^*p* < 0.05, ^∗∗^*p* < 0.01).

Previously, we generated RNA-seq data of wheat young inflorescence at double ridge (DR, Waddington scale W2), floret meristem (FM, W2.5 ∼ 3), anther primordium (AM, W3.5 ∼ 4), and tetrad (TS, W7.5) stages (Feng et al., 2017). We found that 31 homoeologs of 15 TCP genes were present in this dataset (**Supplementary Table S3**). As shown in **Figure 7** and **Supplementary Table S3**, TCP genes with the highest expression level in TS stage were five class II genes, namely *TaPCF5*, *6*, and *TaTCP5*, *18*, and *21*, while the class II gene *TaPCF8* was most highly expressed at the stage W3.5 (**Figure 7**), suggesting that TaTCP genes may be involved in floral organ development. For class II CYC/TB1 genes, *TaTCP24* and *TaTB1-1* showed the lowest expression level at the W7.5 stage, while *TaTCP22* showed similar expression levels from W2.5 to W7.5. For class I PCF genes, *TaPCF3*, *TaTCP9*, and *TaTCP17* were expressed with the highest level at W7.5, while *TaTCP6* was expressed with the highest expression level at DR. Interestingly, *TaTCP7* and *TaTCP28* were constitutively expressed in all the four stages. We studied the expression patterns of wheat TCP genes at more time points using RT-PCR including W5.5, W6.5, and W8.5. The W3.5–W7.5 stages cover the key developmental stages of floral organ initiation and differentiation (Sreenivasulu and Schnurbusch, 2012). The six class II CIN-type TCPs (*TaPCF5*, *6*, *8*, *TaTCP5*, *18*, *21*) were expressed with high levels from W3.5 to W7.5 (**Figure 8**), suggesting that these genes may play important roles in floral organ development. For class II CYC/TB1-type genes, *TaTCP22* showed the highest expression level at W6.5 while *TaTB1-1* was highly expressed at W2 (DR) which was consistent with that of RNA-seq. For six class I PCF-type genes, four (*TaTCP6*, *7*, *17*, and *28*) were consistent with their RNA-seq patterns and two of them (*TaPCF3* and *TaTCP9*) were different, probably caused by the changing of reference genomes for these two detecting methods. For instance, in the old version (Ensemble Triticum_aestivum.IWGSC2.25), only two homoeologs, A and D, were identified for *TaTCP9*, while in the new version (IWGSC RefSeq v1.0), all three homoeologs, A, B, and D, were identified. The change in homoeolog copies may cause inaccurate expression patterns in RNA-seq quantification, different to that of RT-PCR analysis which used primers consensus to all three homoeologs. From W2 to W7.5, the expression level of *TaTCP17* increased significantly (**Figure 8**). Such a pattern may indicate its importance during wheat inflorescence development and should be further studied.

**FIGURE 9:**
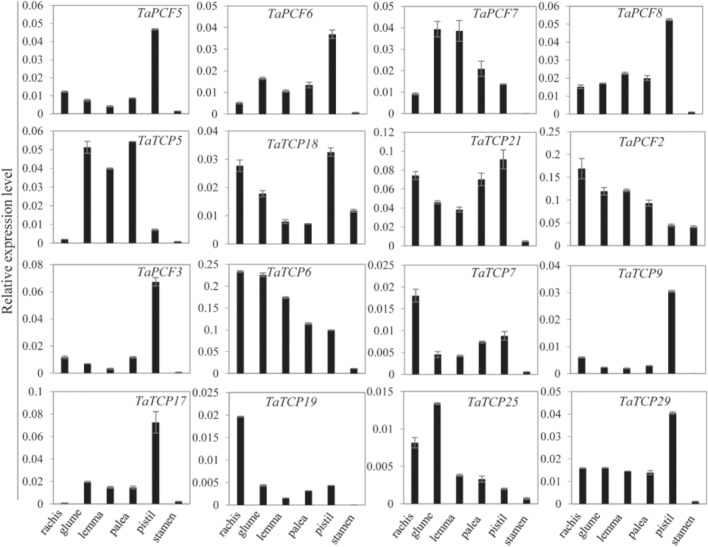
Tissue-specific expressions of wheat TCP genes. Tissues collected from plants at W9.5 and expression levels were detected by qRT-PCR. Three biological duplicates; Y axis represents the relative expression value to *GAPDH* gene, the internal expression control.

**FIGURE 10:**
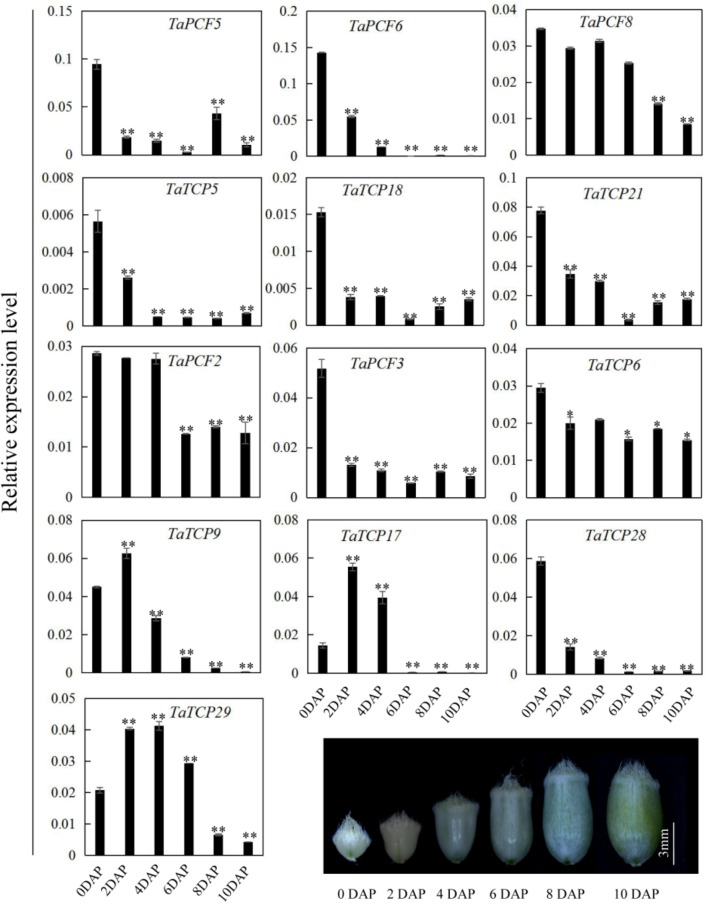
TaTCP gene expression patterns during grain development. Expression levels were detected by qRT-PCR with immature grains at six stages: 0, 2, 4, 6, 8, and 10 days after pollination (DAP). Three biological duplicates were performed; Y axis represents the relative expression value to *GAPDH* gene, the internal control. Asterisks indicate significant difference relative to W2, as determined by Student’s *t*-test (^∗^*p* < 0.05, ^∗∗^*p* < 0.01).

Since most TaTCP genes were expressed at higher levels toward the later stages of floral organ development, we studied their organ-specific expression patterns at the heading date stage (W9.5). A total of six tissues were used including rachis, glume, lemma, palea, pistil, and stamen (**Figure 9**). Two class I genes (*TaTCP7*, *19*) were highly expressed in rachis. Seven TCPs including four class I TCPs (*TaPCF3*, *TaTCP9*, and *17*, *29*) and three class II CIN-type ones (*TaPCF5*, *6*, and *8*) were highly expressed in pistil. To determine whether wheat TCP genes also expressed in immature grain, we detected the expression patterns of the above seven pistil-specific genes in different grain developing stages. Four of them (*TaPCF5*, *6*, *8*, and *3*) were down regulated after pollination which were further down regulated from 2 to 4 DAP (**Figure 10**). Conversely, *TaTCP9* and *TaTCP17* were most highly expressed at 2 DAP, while *TaTCP29* at 2-4 DAP, suggesting that these three genes may play a role in early grain development.

### *In situ* Hybridization Assay of Wheat TCP Genes

**FIGURE 11:**
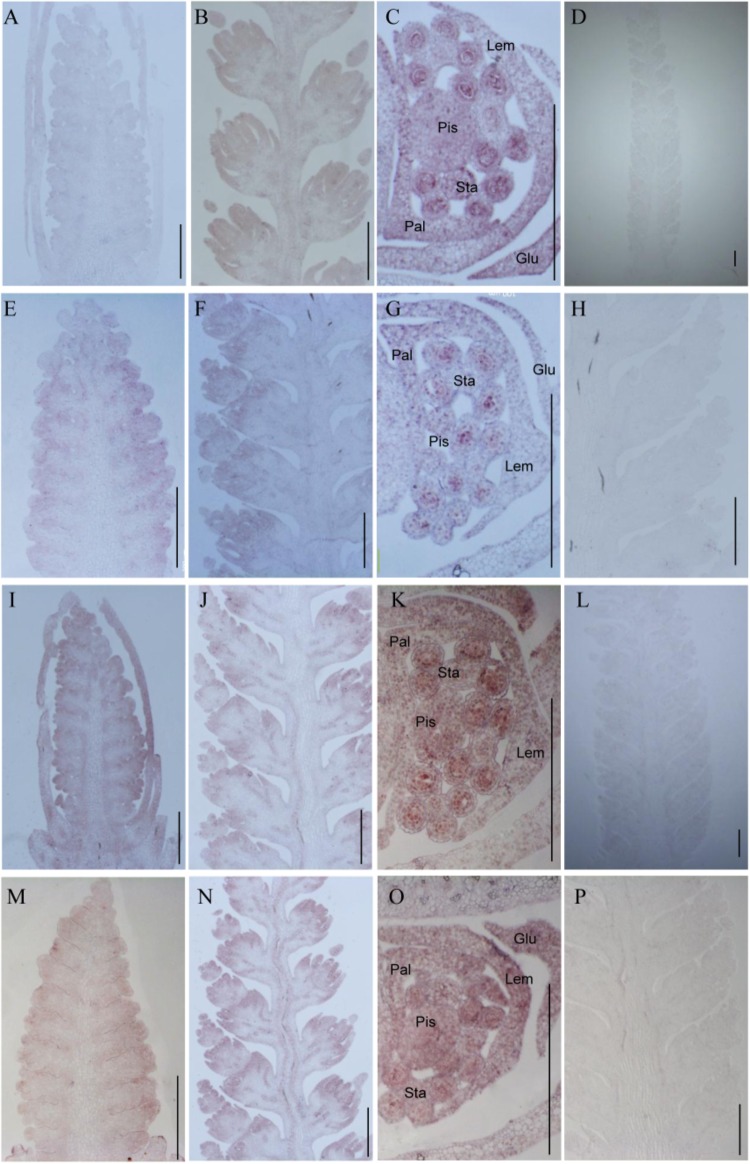
*In situ* hybridization assays of four TaTCP genes. **(A–D)** *TaPCF5*; **(E–H)** *TaPCF6*; **(I–L)** *TaTCP21*; and **(M–P)** *TaTCP18*. Spike developmental stages were W3.5: A, E, I, M; W5.5: B, F, G, N; W6.5: C, G, K, O. Sense probes were used for D, H, L, and P. Glu, glume; Lem, lemma; Pal, palea; Pis, pistil; Sta, stamen; and bar = 500 μm.

To investigate whether TaTCP genes were expressed in specific tissues at early stages of spike development, we conducted *in situ* hybridization of four selected genes with relatively high expression level at W3.5, W5.5, and W6.5. We found that the four CIN-type genes (*TaTCP18*, *21* and *TaPCF5*, *TaPCF6*) were expressed from floret initiation to floral organ maturation and did not display specific expression domains along the spike. As shown in **Figure 11**, the four genes were expressed in multiple floral organs including lemma, palea, pistil, and stamen. The ubiquitous expression of these genes indicates that they may play multiple roles in maintaining cell specificity throughout early inflorescence development.

### Protein–Protein Interaction Patterns Among Wheat TaTCPs

**FIGURE 12:**
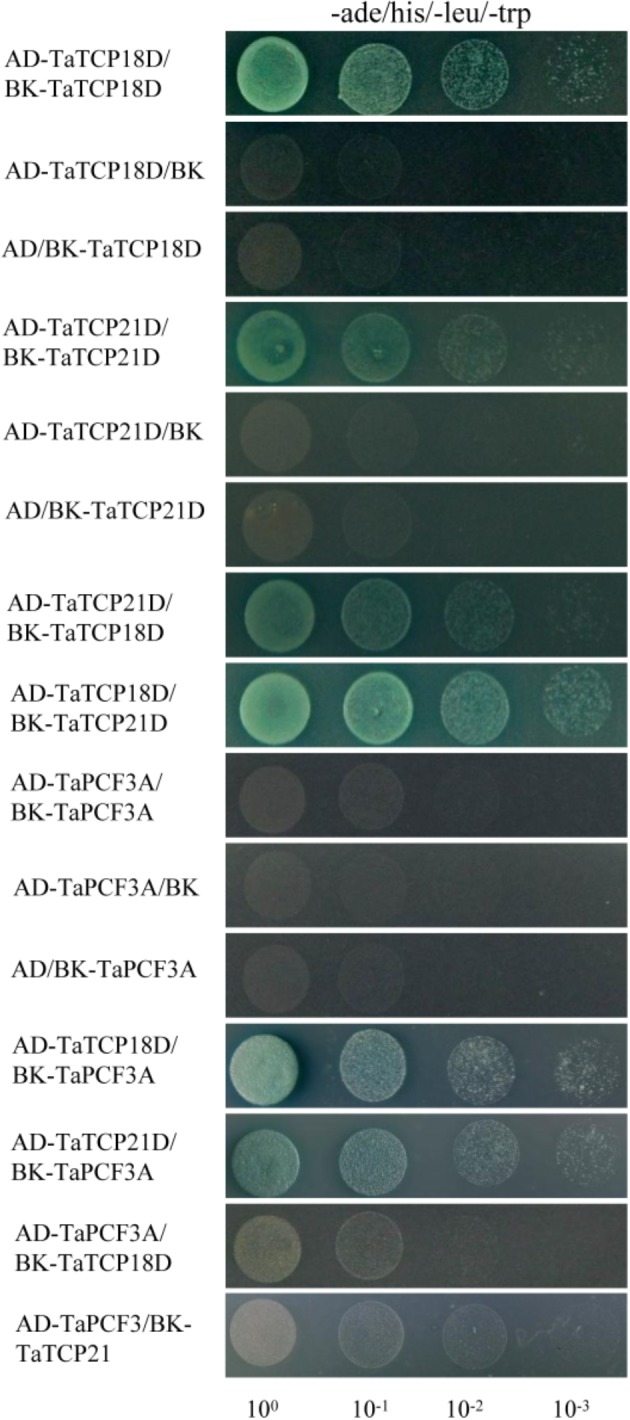
Pair-wise protein-protein interactions among TaTCP18D, TaTCP21D, and TaPCF3A. Coding sequences of TaTCP genes were cloned to pGADT7 (AD) and pGBKT7 (BK) vectors. Interactions among the TaTCP proteins were analyzed by yeast two-hybrid assay. Transformants were assayed for growth on QDO nutritional selection medium.

TCP proteins function by forming protein complexes (Kosugi and Ohashi, 2002; Parapunova et al., 2014). We analyzed the interaction capabilities of several TaTCP proteins not only with relative high expression level but also with the same expression pattern, specifically TaTCP18-D, TaTCP21-D and TaPCF3-A, using yeast two-hybrid assays. These TCP genes were expressed with similar patterns during grain development (**Figure 10**). As shown in **Figure 12** and **Table 4**, class II CIN-type TCPs TaTCP18-D and TaTCP21-D formed both homodimers and heterodimers, while the class I PCF-type TaPCF3-A formed heterodimers with TaTCP18-D or TaTCP21-D but no homodimers were formed. These data indicate that wheat TCP proteins may carry out their functions by interacting with each other to form protein complexes, similar to that in rice and *Arabidopsis*.

**Table 4:** Protein-protein interaction of selected wheat TCP genes.

AD	TaTCP18-D	TaTCP21-D	TaPCF3-A
BD			
TaTCP18-D	✓	✓	✓
TaTCP21-D	✓	✓	✓
TaPCF3-A	X	X	X


*X, no interaction.*

### Characterization of Tetraploid Mutants Carrying Loss-of-Function Alleles of *TaTCP9*

**FIGURE 13:**
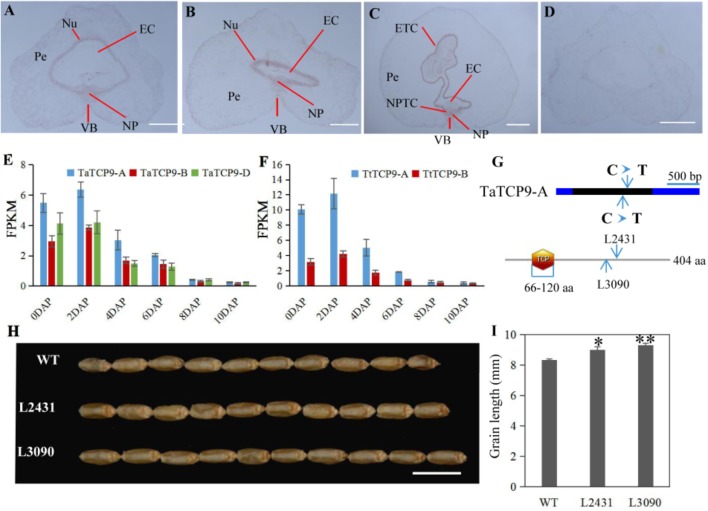
Functional characterization of the wheat *TaTCP9* gene. **(A–D)** In situ hybridization of *TaTCP9* in immature seeds at 2 DAP **(A)**, 4 DAP **(B)**, and 6 DAP **(C)** in Chinese Spring. **(D)** sense control. Pe, percarp; VB, vascular bundle; NP, nucellar projection; EC, endosperm cavity; ETC, endosperm transfer cells; and NPTC, nucellar projection transfer cells. **(E,F)** Expression pattern of *TaTCP9* during grain development in hexaploid wheat **(E)** and tetraploid wheat **(F)** Each point of the FPKM represents the average of three biological replicates. **(G)** the mutation sites on the *TaTCP9-A* gene that gave premature stop codons in two Kronos mutant lines L2431 and L3090. **(H)** grain morphology of the mutants L2431 and L3090 relative to the wild type Kronos. **(I)** Increased grain length in L2431 and L3090. Error bars indicate SD. A total of 23 spikes from six L2341 plants and 12 spikes from four L3090 plants were measured. Student’s *t*-test, ^∗^*p* < 0.05, ^∗∗^*p* < 0.01. Bars in **A–D** indicate 500 μm; Bar in **H** indicates 1 cm.

In rice, *OsTCP19* appears to be an important node in cell signaling which crosslinks stress and developmental pathways (Mukhopadhyay and Tyagi, 2015). *OsTCP9* and *OsTCP19* belong to the same phylogeny branch (**Figure 1A**), but no functional study of *OsTCP9* and its homologs in any other species is available. Here, we firstly studied the tissue specific expression patterns of *TaTCP9* and found that it was highly expressed in pistil at the W9.5 stage (**Figure 9**). A time course study in immature grains showed that its expression peaked at 2 DAP and decreased at later stages of grain development (**Figure 10**), a pattern similar to the rice *TGW6* gene (an IAA-glucose hydrolase) which enhances rice grain weight and increases yield (Ishimaru et al., 2013). To identify the precise expression domain of *TaTCP9*, *in situ* hybridization assays were performed. The results showed that *TaCP9* began to express at early stages of grain development (2, 4, and 6 DAP) and was mainly expressed in endosperm transfer cells (ETC) and nucellar projection transfer cells (NPTCs) (**Figures 13A–D**). These cells are important because the first layer of ETC confers aleurone cell features and the second and third layers of ETC accumulate starch granules and protein bodies (Zheng and Wang, 2011) and actively transport sucrose from photosynthetic tissues to endosperm and embryo (Pfeifer et al., 2014).

We also determined the expression patterns of *TaTCP9* in tetraploid wheat (*Triticum turgidum* ssp. *durum*) because there are mutant lines that are generated using durum variety “Kronos” and can be used to genetically study the function of *TaTCP9* (Uauy et al., 2009; Henry et al., 2014; Krasileva et al., 2017). *TaTCP9* was highly expressed in Kronos grains of 2, 4, and 6 DAP (**Figures 13E,F**). Such a pattern is similar to that in common wheat, indicating that its functions in tetraploid and hexaploid wheat may be conserved. For the two homoeologs in Kronos, *TaTCP9-A* was expressed higher than *TaTCP9-B* (**Figure 13F**). In CS, the A genome homoeolog *TaCTP9-A* also expressed higher than the other two homoeologs *TaTCP9-B* and *TaTCP9-D* (**Figure 13E**). We searched the mutant sequence dataset using genomic sequence of *TaTCP9* and two mutant lines L2341 and L3090 were found to contain C811T and C742T mutations, respectively, on their *TaTCP9-A* homoeolog leading to premature stop codons (**Figure 13G** and **Supplementary Figure S2A**). Although these tetraploid mutants have been selected by their phenotypes for several generations (Krasileva et al., 2017), there are still additional mutation sites in them.

**FIGURE 14:**
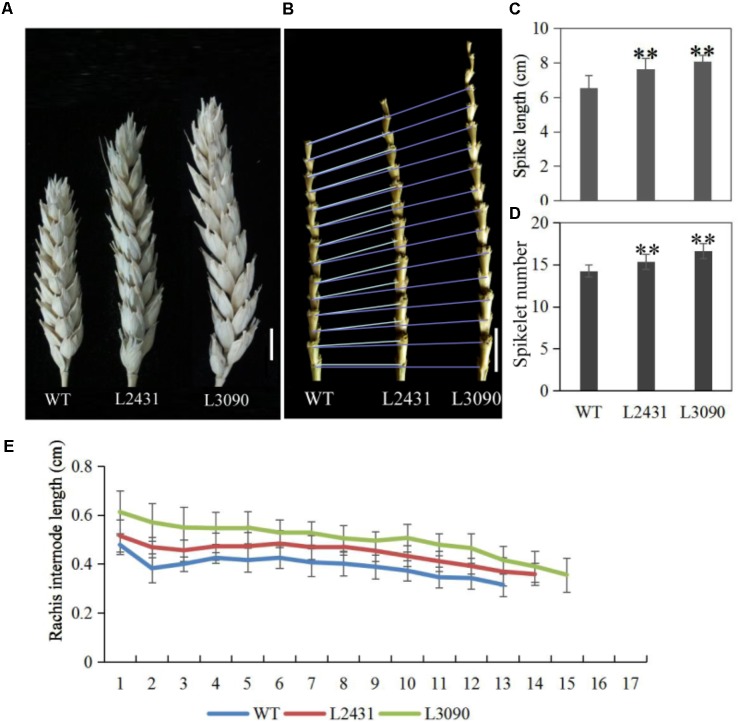
Spike phenotypes of *TaTCP9* mutants in tetraploid Kronos lines. **(A)** spike morphology of the mutants L2431 and L3090 relative to the wild type Kronos. **(B)** Increased internode numbers in L2431 and L3090. **(C,D)** Statistic analysis of spike length and spikelet numbers in L2431 and L3090. **(E)** Comparison of rachis internode lengths in the wild type and the two putative mutant lines L2431 and L3090. Error bars indicate standard deviation (SD). For spike length and spikelet number, 20 spikes from 20 L2341 to L3090 plants were measured. Student’s *t*-test, ^∗^*p* < 0.05, ^∗∗^*p* < 0.01. Bars in **(A,B)** indicate 1 cm.

Morphologically, these two mutant lines showed significantly longer grains than those of the wild type (**Figures 13H,I**). In addition, grain width was wider in L2431 (**Supplementary Figures S2B,C**), resulting in higher 1,000-grain weight (**Supplementary Figure S2D**). Length of spike was also longer for the two mutant lines (**Figures 14A–C**). We found that the increased spike length was caused by both increased spikelet number and increased rachis internode length (**Figures 14D,E**).

Previous work showed that cell size and number increase most significantly in spikes from W2.5 to W3.5 (Feng et al., 2017). We then looked for all mutated genes (those with stop gain and altered splicing) for their expression patterns between these two stages (**Supplementary Datasets S1**–**S4**), particularly those that may be relevent to this process, such as those fell in the MapMan bins of cell cycle and devision, organization (RNA processing), recognition, and transcription, hormone, development (**Supplementary Tables S4**–**S7**). As shown in **Supplementary Figures S3**–**S6**, only two genes *TaTCP9* (Traes_6AL_DA27ABCA61) and Traes_1BS_8A19C460B (an Auxin-responsive Aux/IAA gene) displayed a significant expression increase between W2.5 to W3.5, with the former having stop gain mutations in both L2431 and L3090 and the latter having such a mutation only in L2431. These data suggest that wheat *TaTCP9* may indeed play a role in wheat grain development, although additional genetic study of these mutant lines regarding the function of *TaTCP9* should be further performed.

## Discussion

TCPs govern key plant developmental processes and have profound effects on meristem patterning and organ differentiation, often by mediating hormone mechanisms (Nicolas and Cubas, 2016). The polyploid nature of common wheat renders expansion of the TCP family in this important crop. Subsequent subfunctionalization of these homoeologs and more versatile gene dosages may contribute to the enhanced adaptation of wheat. In wheat, the general organization of the TCP family remained well conserved with significantly more members in the class I subfamily than those in the class II (Navaud et al., 2007).

### The Duplication of Wheat *TaTB1*, an Important Tillering Gene

The importance of TCP genes were firstly demonstrated by the maize *TB1* gene for its role in maize domestication as a negative regulator for the growth of axillary buds and hence branching (Doebley et al., 1995). The process may represent a common mechanism for post-embryonic development of the primary shoot architecture. Phytohormones, especially auxin and cytokinin, play pivotal roles during lateral branch development. The rice *TB1* ortholog *OsTB1* (or *FINE CULM1*, *FC1*) and the *Arabidopsis BRANCHED 1* (*BRC1*) appear to play similar roles by negatively regulating lateral branching (Doebley et al., 1997; Takeda et al., 2003; Aguilar-Martinez et al., 2007). Moreover, *OsTB1* is directly regulated by *IPA1/OsSPL14* to suppress tillering in rice (Lu et al., 2013).

For wheat, in addition to the increased copy number of *TB1* homoeologs due to polyploidization, it is duplicated within each subgenome, with a second copy found on chromosome 5, in addition to the orthologous one on chromosome 4. This is in contrast to rice, Brachypodium, and *Arabidopsis* in which only one TB1 gene is present. The orthologous chromosome 4 *TaTB1-1* was shown recently as a key regulator for wheat inflorescence and plant architecture. It interacts with the Flowering Locus T1 (FT1) to regulate spikelet development (Dixon et al., 2018). The second copy of *TaTB1* (*TaTB1-2*) had a different expression pattern from *TaTB1-1* with the former having highest expression level at W6.5 when most floral organs were under development and the latter having its maximum expression level at the DR stage (W2.0). The subfunctionalization of *TaTB1-2* is evident and should be further studied.

### Nearly All Wheat TCP Genes Are Triplet With Three Homoeologs

Common wheat is a hexaploid (AABBDD, 2n = 6x = 42) that arose 7000–12,000 years ago following the hybridization of the early-domesticated allotetraploid *T. turgidum* ssp. *dicoccon* (AABB) and the diploid goat grass *Aegilops tauschii* (DD, 2n = 2× = 14) (Kihara, 1944; McFadden and Sears, 1946; Huang et al., 2002; Matsuoka, 2011). Thus, most genes are expected to have three homoeoalleles although in many cases the three homoeoalleles are not equally transcribed (Shitsukawa et al., 2007; Geng et al., 2013; Hu et al., 2013). Despite this, among the chromosome survey sequence (CSS) less than 23.16% genes (6576 triplets totaling 19,728 genes out of 85,173 high-confidence gene models) were identified as triplets, loci with three homoeologs, using a reciprocal best-hits approach with BLASTP *e*-value <= 1e-5 and identity > = 90% (Pfeifer et al., 2014).

Interestingly, all wheat TCP genes conferred three homoeologs indicating that they were preferentially retained after two rounds of polyploidization. *Cis*-element analysis showed clear differences between many homoeologs, consistent with their differential expression patterns in RNA-seq data. The maintenance of TCP homoeologs indicate they are under purifying selection while the divergence in *cis*-element patterns suggests on-going subfunctionalization of wheat homoeologs.

### TCP Genes are Ubiquitously Expressed During Early Spike Development in Wheat

The profound functions of TCP genes from meristem maintenance to organ development require the expression of these genes in various tissues and organs. In this study, we focused on the expression patterns of wheat TCPs in spike and grain development. The relative low expression levels of wheat TCP genes are consistent with those identified in the orchid (De Paolo et al., 2015). For some wheat TCP genes, no signal can be detected by *in situ* hybridization. However, the expression range of wheat TCPs covers the whole processes of young spike and immature grain development. Such patterns indicate wide functions of this gene family in multiple aspects of wheat development.

Despite their versatile functions in plant development, to our knowledge, TCPs are rarely reported to be involved in grain development. We present preliminary data that wheat *TaTCP9* gene might be involved in spike and grain development. *TaTCP9* was expressed highly in pistil at the W9.5 stage and in immature grains at 2 DAP. *In situ* hybridization showed that *TaTCP9* expressed in ETC and NPTCs. In *Arabidopsis*, as the key step to achieve final size and function for cells, endoreduplication is prevalent during plant development. *AtTCP15*, for instance, plays an important role in regulating endoreduplication during *Arabidopsis* development (Li et al., 2012). Moreover, mutants in two related class I TCP genes display a range of growth-related phenotypes, consistent with their dynamic expression patterns. The two genes influence plant stature by promoting cell division in young internodes (Kieffer et al., 2011). In our study, the increased spikelet number as well as increased rachis internode length may be accounted for in mutant plants. It is probable that the mutation in *TaTCP9* might increase both cell number and cell size. Together with the phenotypes of the tetraploid mutants, we propose that *TaTCP9* is highly possible to be involved in grain development and hence final grain yield, although further study is needed to reach a final conclusion.

Like TCPs in other plants, most wheat TCP genes expressed in multiple tissues and developmental stages, indicating their important roles in wheat development. Further molecular study of these genes should reveal more functional mechanisms for these genes. Since wheat is a polyploid, different to other major crops, study of wheat TCPs may help further understanding dosage-dependent working modes in this polyploid plant and may contribute to genetic engineering for wheat yield improvement.

## Author Contributions

AL and LM planned and designed the research. JZ, YL, SG, GaS, MJ, FW, GuS, NF, XK, and LC performed the experiments. ZZ, JZ, YL, and JG analyzed the data. AL, LM, and JZ wrote the article, with contributions from all the authors.

## Conflict of Interest Statement

The authors declare that the research was conducted in the absence of any commercial or financial relationships that could be construed as a potential conflict of interest. The reviewer NP and the handling Editor declared their shared affiliation.

## Footnotes

1 https://urgi.versailles.inra.fr/download/iwgsc/IWGSC_RefSeq_Assemblies/v1.0/

2 http://smart.embl-heidelberg.de/

3 http://www.expasy.org/tools/

4 http://meme-suite.org/

5 https://urgi.versailles.inra.fr/download/iwgsc/IWGSC_RefSeq_Assemblies/v1.0/

## Supplementary Material

FIGURE S1 Protein motifs of TaTCPs. Each color represents a specific motif in the protein identified using the MEME motif search tool. The order represents the relative locations of motifs in a protein sequence and does not represent the actual location and size.

FIGURE S2 Expression patterns of spike development genes in the mutant L2431 with stop-gain mutations. Genes belong to four MapMan functional bins (listed in Supplementary Tables S3–S6) including cell cycle and division, transcription, hormone, and development and contained stop_gain mutation sites in L2431. RNA-seq data were from Feng et al. (2017).

FIGURE S3 Expression patterns of spike development genes in the mutant L2431 with alternative splicing mutations.

FIGURE S4 Expression patterns of spike development genes in the mutant L3090 with stop-gain mutations.

FIGURE S5 Expression patterns of spike development genes in the mutant L3090 with alternative splicing mutations.

FIGURE S6 The effect of mutations of *TaTCP9* on grain development. **(A)** Partial sequence alignment of *TaTCP9* from wild type (WT) and two mutants L2431 and L3090, showing C811T in L2341 and C742T in L3090. **(B)** Grain width comparison between the mutants L2431 and L3090 and the wild type Kronos. **(C,D)** Statistic analysis of grain width **(C)** and thousand-grain weight (TGW) **(D)** in L2431 and L3090. Error bars indicate standard errors. A total of 23 spikes from six L2341 plants and 12 spikes from four L3090 plants were measured. Student’s t-test, ^∗∗^*p* < 0.01. Bar in panel B indicates 1 cm.

DATASET S1 Stop-gained mutation sites list of the Kronos mutant L2431.

DATASET S2 Splice mutation sites in the Kronos mutant L2431.

DATASET S3 Stop-gained mutation sites list in the Kronos mutant L3090.

DATASET S4 Splice mutation sites in the Kronos mutant L3090.

TABLE S1 List of primer used in this study.

TABLE S2 Gene sequence ID used in **Figure 2** and **Table 2**.

TABLE S3 Correspondence of *TaTCP* gene models in the old and the new reference genomes.

TABLE S4 List of important genes with stop_gained mutant sites in the Kronos mutant L3090.

TABLE S5 List of important genes with splicing mutation sites in the Kronos mutant L3090.

TABLE S6 List of important genes with stop_gained mutant sites in the Kronos mutant L2431.

TABLE S7 List of important genes with splicing mutation sites in the Kronos mutant L2431.

## References

[B1] Aguilar-Martinez J. A. Poza-Carrion C. Cubas P. (2007). *Arabidopsis* branched1 acts as an integrator of branching signals within axillary buds. *Plant Cell* 19 458–472. 10.1105/tpc.106.048934 17307924 PMC1867329

[B2] Almeida D. M. Gregorio G. B. Oliveira M. M. Saibo N. J. (2017). Five novel transcription factors as potential regulators of OsNHX1 gene expression in a salt tolerant rice genotype. *Plant Mol. Biol.* 93 61–77. 10.1007/s11103-016-0547-7 27766460

[B3] Anderson S. L. Teakle G. R. Martino-Catt S. J. Kay S. A. (1994). Circadian clock- and phytochrome-regulated transcription is conferred by a 78 bp *cis*-acting domain of the *Arabidopsis* CAB2 promoter. *Plant J.* 6 457–470. 10.1046/j.1365-313x.1994.6040457.x 7987408

[B4] Berkman P. J. Skarshewski A. Manoli S. Lorenc M. T. Stiller J. Smits L. (2012). Sequencing wheat chromosome arm 7BS delimits the 7BS/4AL translocation and reveals homoeologous gene conservation. *Theor. Appl. Genet.* 124 423–432. 10.1007/s00122-011-1717-2 22001910

[B5] Bobb A. J. Chern M. S. Bustos M. M. (1997). Conserved RY-repeats mediate transactivation of seed-specific promoters by the developmental regulator PvALF. *Nucleic Acids Res.* 25 641–647. 10.1093/nar/25.3.641 9016607 PMC146457

[B6] Chai W. Jiang P. Huang G. Jiang H. Li X. (2017). Identification and expression profiling analysis of TCP family genes involved in growth and development in maize. *Physiol. Mol. Biol. Plants* 23 779–791. 10.1007/s12298-017-0476-1 29158628 PMC5671458

[B7] Clark R. M. Wagler T. N. Quijada P. Doebley J. (2006). A distant upstream enhancer at the maize domestication gene tb1 has pleiotropic effects on plant and inflorescent architecture. *Nat. Genet.* 38 594–597. 10.1038/ng1784 16642024

[B8] Cubas P. Lauter N. Doebley J. Coen E. (1999). The TCP domain: a motif found in proteins regulating plant growth and development. *Plant J.* 18 215–222. 10.1046/j.1365-313X.1999.00444.x 10363373

[B9] Danisman S. (2016). TCP transcription factors at the interface between environmental challenges and the plant’s growth responses. *Front. Plant Sci.* 7:1930. 10.3389/fpls.2016.01930 28066483 PMC5174091

[B10] De Paolo S. Gaudio L. Aceto S. (2015). Analysis of the TCP genes expressed in the inflorescence of the orchid *Orchis italica*. *Sci. Rep.* 5:16265. 10.1038/srep16265 26531864 PMC4632031

[B11] Devos K. M. Dubcovsky J. Dvorak J. Chinoy C. N. Gale M. D. (1995). Structural evolution of wheat chromosomes 4A, 5A, and 7B and its impact on recombination. *Theor. Appl. Genet.* 91 282–288. 10.1007/BF00220890 24169776

[B12] Dixon L. E. Greenwood J. R. Bencivenga S. Zhang P. Cockram J. Mellers G. (2018). Teosinte branched1 regulates inflorescence architecture and development in bread wheat (*Triticum aestivum* L.). *Plant Cell* 30 563–581. 10.1105/tpc.17.00961 29444813 PMC5894836

[B13] Doebley J. Stec A. Gustus C. (1995). teosinte branched1 and the origin of maize: evidence for epistasis and the evolution of dominance. *Genetics* 141 333–346. 8536981 10.1093/genetics/141.1.333 PMC1206731

[B14] Doebley J. Stec A. Hubbard L. (1997). The evolution of apical dominance in maize. *Nature* 386 485–488. 10.1038/386485a0 9087405

[B15] Feng N. Song G. Guan J. Chen K. Jia M. Huang D. (2017). Transcriptome profiling of wheat inflorescence development from spikelet initiation to floral patterning identified stage-specific regulatory genes. *Plant Physiol.* 174 1779–1794. 10.1104/pp.17.00310 28515146 PMC5490901

[B16] Francis A. Dhaka N. Bakshi M. Jung K. H. Sharma M. K. Sharma R. (2016). Comparative phylogenomic analysis provides insights into TCP gene functions in Sorghum. *Sci. Rep.* 6:38488. 10.1038/srep38488 27917941 PMC5137041

[B17] Geng S. Li A. Tang L. Yin L. Wu L. Lei C. (2013). TaCPK2-A, a calcium-dependent protein kinase gene that is required for wheat powdery mildew resistance enhances bacterial blight resistance in transgenic rice. *J. Exp. Bot.* 64 3125–3136. 10.1093/jxb/ert146 23918959 PMC3733141

[B18] Henry I. M. Nagalakshmi U. Lieberman M. C. Ngo K. J. Krasileva K. V. Vasquez-Gross H. (2014). Efficient genome-wide detection and cataloging of EMS-induced mutations using exome capture and next-generation sequencing. *Plant Cell* 26 1382–1397. 10.1105/tpc.113.121590 24728647 PMC4036560

[B19] Hu Z. Han Z. Song N. Chai L. Yao Y. Peng H. (2013). Epigenetic modification contributes to the expression divergence of three TaEXPA1 homoeologs in hexaploid wheat (*Triticum aestivum*). *New Phytol.* 197 1344–1352. 10.1111/nph.12131 23360546

[B20] Huang S. Sirikhachornkit A. Su X. Faris J. Gill B. Haselkorn R. (2002). Genes encoding plastid acetyl-CoA carboxylase and 3-phosphoglycerate kinase of the *Triticum*/*Aegilops* complex and the evolutionary history of polyploid wheat. *Proc. Natl. Acad. Sci. U.S.A.* 99 8133–8138. 10.1073/pnas.072223799 12060759 PMC123033

[B21] Ishimaru K. Hirotsu N. Madoka Y. Murakami N. Hara N. Onodera H. (2013). Loss of function of the IAA-glucose hydrolase gene TGW6 enhances rice grain weight and increases yield. *Nat. Genet.* 45 707–711. 10.1038/ng.2612 23583977

[B22] Kieffer M. Master V. Waites R. Davies B. (2011). TCP14 and TCP15 affect internode length and leaf shape in *Arabidopsis.* *Plant J.* 68 147–158. 10.1111/j.1365-313X.2011.04674.x 21668538 PMC3229714

[B23] Kihara H. (1944). Discovery of the DD analyser, one of the tetraploid. *Agric. Hortic.* 19 889–890.

[B24] Kim J. K. Cao J. Wu R. (1992). Regulation and interaction of multiple protein factors with the proximal promoter regions of a rice high pI alpha-amylase gene. *Mol. Gen. Genet.* 232 383–393. 10.1007/bf00266241 1375314

[B25] Kosugi S. Ohashi Y. (1997). PCF1 and PCF2 specifically bind to cis elements in the rice proliferating cell nuclear antigen gene. *Plant Cell* 9 1607–1619. 10.1105/tpc.9.9.1607 9338963 PMC157037

[B26] Kosugi S. Ohashi Y. (2002). DNA binding and dimerization specificity and potential targets for the TCP protein family. *Plant J.* 30 337–348. 10.1046/j.1365-313X.2002.01294.x 12000681

[B27] Krasileva K. V. Vasquez-Gross H. A. Howell T. Bailey P. Paraiso F. Clissold L. (2017). Uncovering hidden variation in polyploid wheat. *Proc. Natl. Acad. Sci. U.S.A.* 114 E913–E921. 10.1073/pnas.1619268114 28096351 PMC5307431

[B28] Larkin M. A. Blackshields G. Brown N. P. Chenna R. McGettigan P. A. McWilliam H. (2007). Clustal wand clustal X version 2.0. *Bioinformatics* 23 2947–2948. 10.1093/bioinformatics/btm404 17846036

[B29] Lescot M. Déhais P. Thijs G. Marchal K. Moreau Y. Van de Peer Y. (2002). PlantCARE, a database of plant *cis*-acting regulatory elements and a portal to tools for *in silico* analysis of promoter sequences. *Nucleic Acids Res.* 30 325–327. 10.1093/nar/30.1.325 11752327 PMC99092

[B30] Li Z. Y. Li B. Dong A. W. (2012). The *Arabidopsis* transcription factor AtTCP15 regulates endoreduplication by modulating expression of key cell-cycle genes. *Mol. Plant* 5 270–280. 10.1093/mp/ssr086 21992944

[B31] Liu C. Teo Z. W. Bi Y. Song S. Xi W. Yang X. (2013). A conserved genetic pathway determines inflorescence architecture in *Arabidopsis* and rice. *Dev. Cell* 24 612–622. 10.1016/j.devcel.2013.02.013 23537632

[B32] Liu J. Cheng X. Liu P. Sun J. (2017). miR156-targeted SBP-Box transcription factors interact with DWARF53 to regulate teosinte branched1 and barren stalk1 expression in bread wheat. *Plant Physiol.* 174 1931–1948. 10.1104/pp.17.00445 28526703 PMC5490914

[B33] Lu Z. Yu H. Xiong G. Wang J. Jiao Y. Liu G. (2013). Genome-wide binding analysis of the transcription activator ideal plant architecture1 reveals a complex network regulating rice plant architecture. *Plant Cell* 25 3743–3759. 10.1105/tpc.113.113639 24170127 PMC3877814

[B34] Luo D. Carpenter R. Vincent C. Copsey L. Coen E. (1996). Origin of floral asymmetry in *Antirrhinum*. *Nature* 383 794–799. 10.1038/383794a0 8893002

[B35] Martín-Trillo M. Cubas P. (2010). TCP genes: a family snapshot ten years later. *Trends Plant Sci.* 15 31–39. 10.1016/j.tplants.2009.11.003 19963426

[B36] Matsuoka Y. (2011). Evolution of polyploid *Triticum* wheats under cultivation: the role of domestication, natural hybridization and allopolyploid speciation in their diversification. *Plant Cell Physiol.* 52 750–764. 10.1093/pcp/pcr018 21317146

[B37] McFadden E. S. Sears E. R. (1946). The origin of *Triticum spelta* and its free-threshing hexaploid relatives. *J. Hered.* 37 107–116. 20985728 10.1093/oxfordjournals.jhered.a105590

[B38] Mondragon-Palomino M. Trontin C. (2011). High time for a roll call: gene duplication and phylogenetic relationships of TCP-like genes in monocots. *Ann. Bot.* 107 1533–1544. 10.1093/aob/mcr059 21444336 PMC3108806

[B39] Mukhopadhyay P. Tyagi A. K. (2015). OsTCP19 influences developmental and abiotic stress signaling by modulating ABI4-mediated pathways. *Sci. Rep.* 5:9998. 10.1038/srep09998 25925167 PMC4415230

[B40] Murre C. McCaw P. S. Baltimore D. (1989). A new DNA binding and dimerization motif in immunoglobulin enhancer binding, daughterless. MyoD, and myc proteins. *Cell* 56 777–783. 2493990 10.1016/0092-8674(89)90682-x

[B41] Nag A. King S. Jack T. (2009). miR319a targeting of TCP4 is critical for petal growth and development in *Arabidopsis.* *Proc. Natl. Acad. Sci. U.S.A.* 106 22534–22539. 10.1073/pnas.0908718106 20007771 PMC2799693

[B42] Navaud O. Dabos P. Carnus E. Tremousaygue D. Herve C. (2007). TCP transcription factors predate the emergence of land plants. *J. Mol. Evol.* 65 23–33. 10.1007/s00239-006-0174-z 17568984

[B43] Nicolas M. Cubas P. (2016). TCP factors: new kids on the signaling block. *Curr. Opin. Plant Biol.* 33 33–41. 10.1016/j.pbi.2016.05.006 27310029

[B44] Ori N. Cohen A. R. Etzioni A. Brand A. Yanai O. Shleizer S. (2007). Regulation of LANCEOLATE by miR319 is required for compound-leaf development in tomato. *Nat. Genet.* 39 787–791. 10.1038/ng2036 17486095

[B45] Palatnik J. F. Allen E. Wu X. Schommer C. Schwab R. Carrington J. C. (2003). Control of leaf morphogenesis by miRNAs. *Nature* 425 257–263. 10.1038/nature01958 12931144

[B46] Parapunova V. Busscher M. Busscher-Lange J. Lammers M. Karlova R. Bovy A. G. (2014). Identification, cloning and characterization of the tomato TCP transcription factor family. *BMC Plant Biol.* 14:157. 10.1186/1471-2229-14-157 24903607 PMC4070083

[B47] Pfeifer M. Kugler K. G. Sandve S. R. Zhan B. Rudi H. Hvidsten T. R. (2014). Genome interplay in the grain transcriptome of hexaploid bread wheat. *Science* 345:1250091. 10.1126/science.1250091 25035498

[B48] Rouster J. Leah R. Mundy J. Cameron-Mills V. (1997). Identification of a methyl jasmonate-responsive region in the promoter of a lipoxygenase 1 gene expressed in barley grain. *Plant J.* 11 513–523. 10.1046/j.1365-313x.1997.11030513.x 9107039

[B49] Sharma R. Kapoor M. K. Tyagi A. Kapoor S. (2010). Comparative transcript profiling of TCP family genes provide insight into gene functions and diversification in rice and *Arabidopsis.* *J. Plant Mol. Biol. Biotechnol.* 1 24–38.

[B50] Shen Q. Ho T. H. (1995). Functional dissection of an abscisic acid (ABA)-inducible gene reveals two independent ABA-responsive complexes each containing a G-box and a novel *cis*-acting element. *Plant Cell* 7 295–307. 10.1105/tpc.7.3.295 7734964 PMC160783

[B51] Shitsukawa N. Tahira C. Kassai K. Hirabayashi C. Shimizu T. Takumi S. (2007). Genetic and epigenetic alteration among three homoeologous genes of a class E MADS box gene in hexaploid wheat. *Plant Cell* 19 1723–1737. 10.1105/tpc.107.051813 17586655 PMC1955732

[B52] Sreenivasulu N. Schnurbusch T. (2012). A genetic playground for enhancing grain number in cereals. *Trends Plant Sci.* 17 91–101. 10.1016/j.tplants.2011.11.003 22197176

[B53] Takeda T. Suwa Y. Suzuki M. Kitano H. Ueguchi-Tanaka M. Ashikari M. (2003). The OsTB1 gene negatively regulates lateral branching in rice. *Plant J.* 33 513–520. 10.1046/j.1365-313X.2003.01648.x 12581309

[B54] Uauy C. Paraiso F. Colasuonno P. Tran R. K. Tsai H. Berardi S. (2009). A modified TILLING approach to detect induced mutations in tetraploid and hexaploid wheat. *BMC Plant Biol.* 9:115. 10.1186/1471-2229-9-115 19712486 PMC2748083

[B55] Ulmasov T. Murfett J. Hagen G. Guilfoyle T. J. (1997). Aux/IAA proteins repress expression of reporter genes containing natural and highly active synthetic auxin response elements. *Plant Cell* 9 1963–1971. 10.1105/tpc.9.11.1963 9401121 PMC157050

[B56] Waddington S. R. Cartwright P. M. Wall P. C. (1983). A quantitative scale of spike initial and pistil development in barley and wheat. *Ann. Bot.* 51 119–130. 10.1093/oxfordjournals.aob.a086434

[B57] Wang R. L. Stec A. Hey J. Lukens L. Doebley J. (1999). The limits of selection during maize domestication. *Nature* 398 236–239. 10.1038/18435 10094045

[B58] Wang W. Liu J. H. (2015). Genome-wide identification and expression analysis of the polyamine oxidase gene family in sweet orange (*Citrus sinensis*). *Gene* 555 421–429. 10.1016/j.gene.2014.11.042 25445392

[B59] Washida H. Wu C. Y. Suzuki A. Yamanouchi U. Akihama T. Harada K. (1999). Identification of *cis*-regulatory elements required for endosperm expression of the rice storage protein glutelin gene GluB-1. *Plant Mol. Biol.* 40 1–12. 10.1023/a:1026459229671 10394940

[B60] Yao X. Ma H. Jian Wang J. Zhang D. (2007). Genome-wide comparative analysis and expression pattern of TCP gene families in *Arabidopsis thaliana* and *Oryza sativa*. *J. Integr. Plant Biol.* 49 885–897. 10.1111/j.1744-7909.2007.00509.x

[B61] Yuan Z. Gao S. Xue D. W. Luo D. Li L. T. Ding S. Y. (2009). RETARDED PALEA1 controls palea development and floral zygomorphy in rice. *Plant Physiol.* 149 235–244. 10.1104/pp.108.128231 18952859 PMC2613737

[B62] Zhang C. Ding Z. Wu K. Yang L. Li Y. Yang Z. (2016). Suppression of jasmonic acid-mediated defense by viral-inducible MicroRNA319 facilitates virus infection in rice. *Mol. Plant* 9 1302–1314. 10.1016/j.molp.2016.06.014 27381440

[B63] Zhao T. Xia H. Liu J. Ma F. (2014). The gene family of dehydration responsive element-binding transcription factors in grape (*Vitis vinifera*): genome-wide identification and analysis, expression profiles, and involvement in abiotic stress resistance. *Mol. Biol. Rep.* 41 1577–1590. 10.1007/s11033-013-3004-6 24402876

[B64] Zheng Y. Wang Z. (2011). Contrast observation and investigation of wheat endosperm transfer cells and nucellar projection transfer cells. *Plant Cell Rep.* 30 1281–1288. 10.1007/s00299-011-1039-5 21359829

