# Supplementary figures and images for: Genome-Wide Identification and Expression Profiling of the TCP Family Genes in Spike and Grain Development of Wheat (Triticum aestivum L.)
Source: Front Plant Sci. 2018 Sep 10;9:1282. doi: 10.3389/fpls.2018.01282 (PMC6160802; doi:10.3389/fpls.2018.01282)

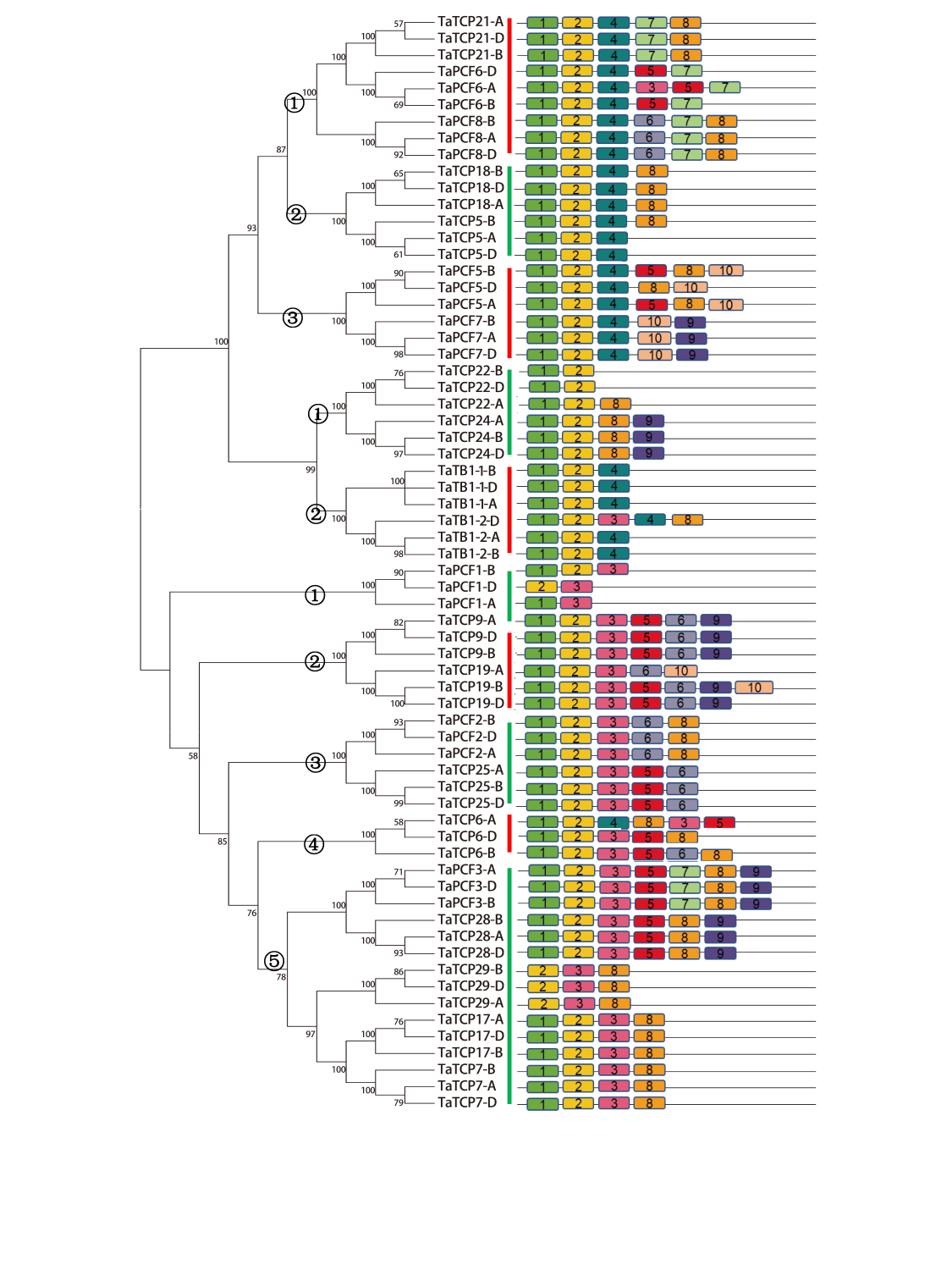

Supplement: FIGURE S1 — Protein motifs of TaTCPs. Each color represents a specific motif in the protein identified using the MEME motif search tool. The order represents the relative locations of motifs in a protein sequence and does not represent the actual location and size. [file Image_1.tif]

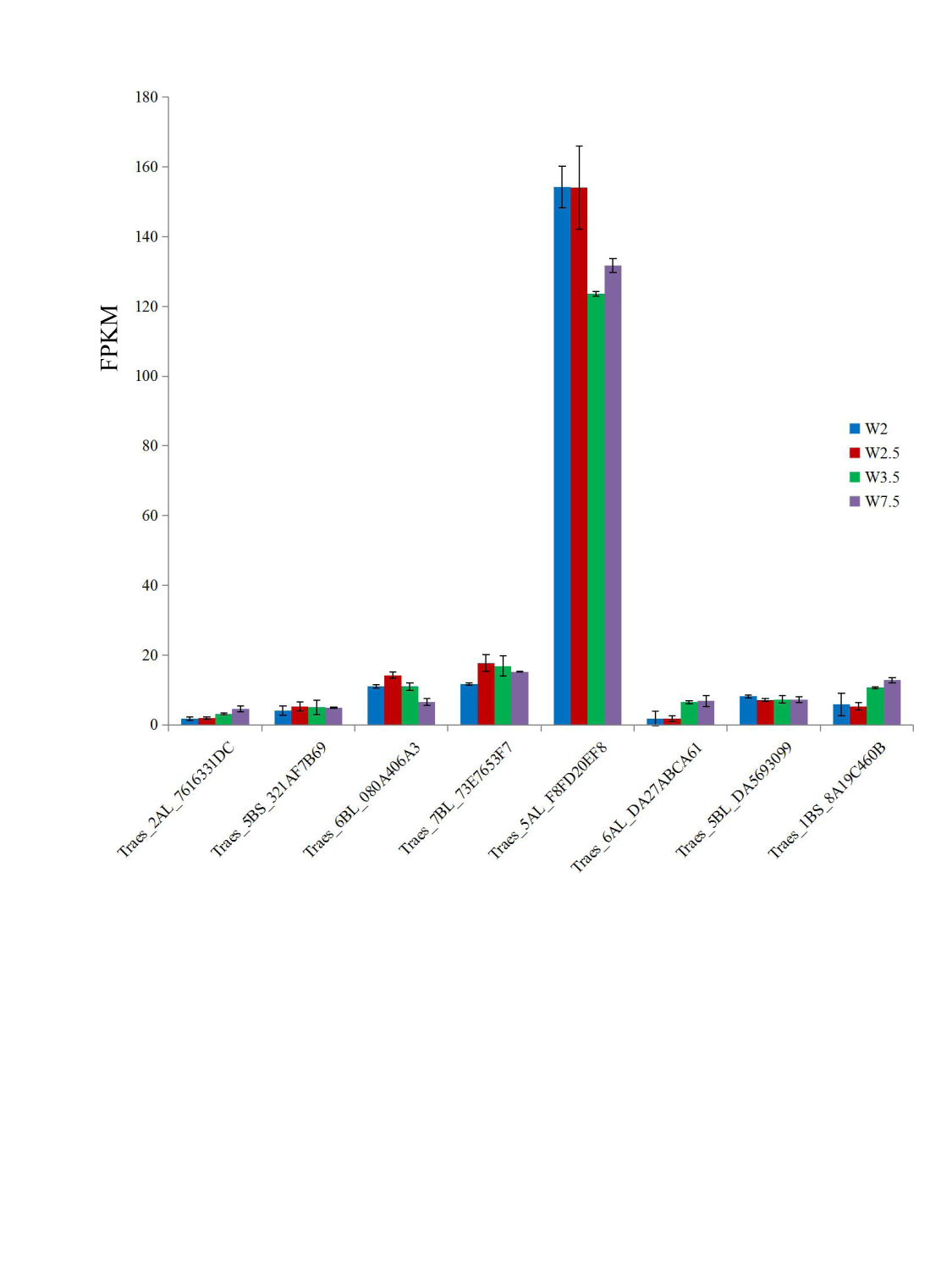

Supplement: FIGURE S2 — Expression patterns of spike development genes in the mutant L2431 with stop-gain mutations. Genes belong to four MapMan functional bins (listed in Supplementary Tables S3–S6) including cell cycle and division, transcription, hormone, and development and contained stop_gain mutation sites in L2431. RNA-seq data were from Feng et al. (2017). [file Image_2.tif]

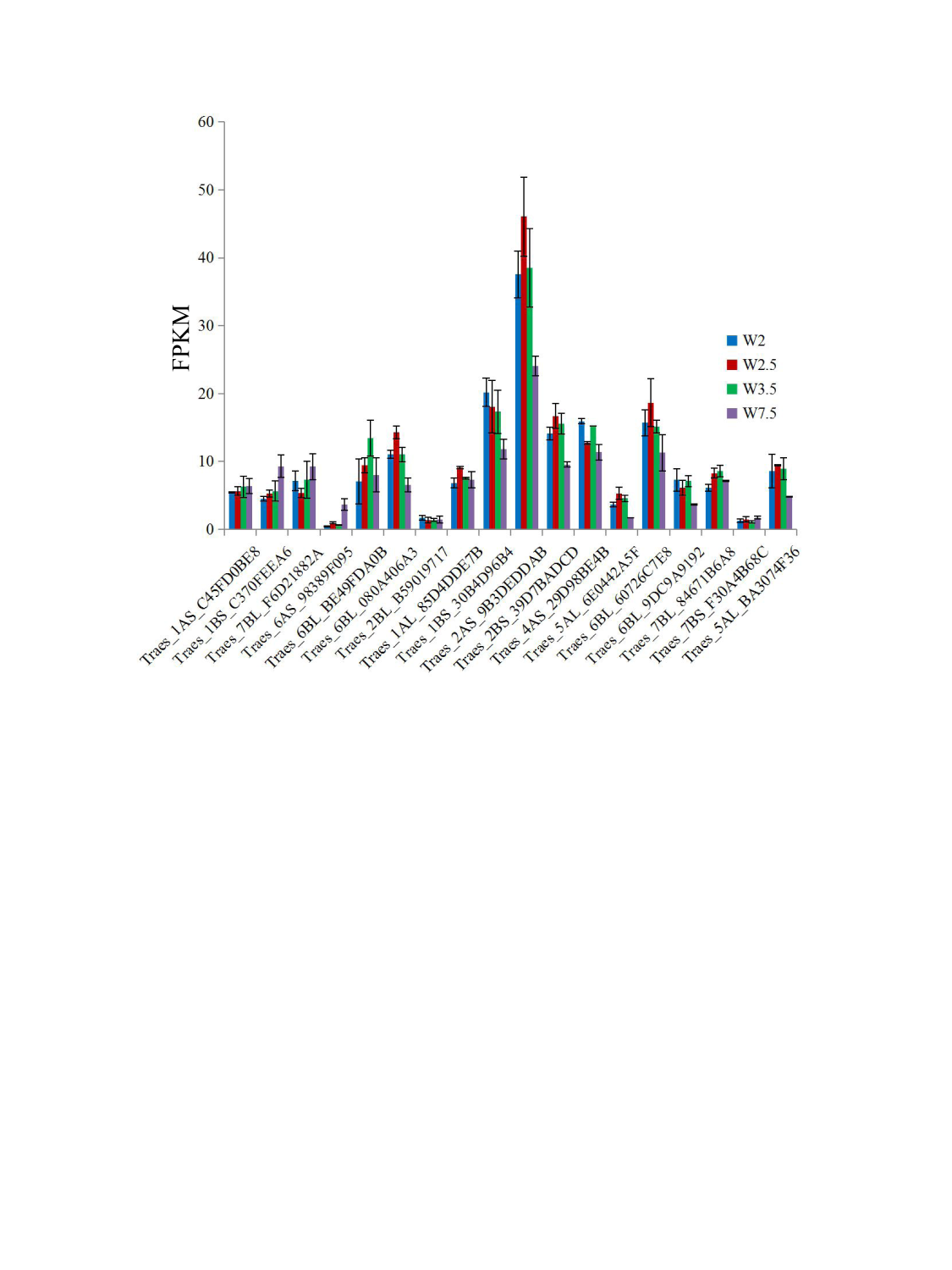

Supplement: FIGURE S3 — Expression patterns of spike development genes in the mutant L2431 with alternative splicing mutations. [file Image_3.tif]

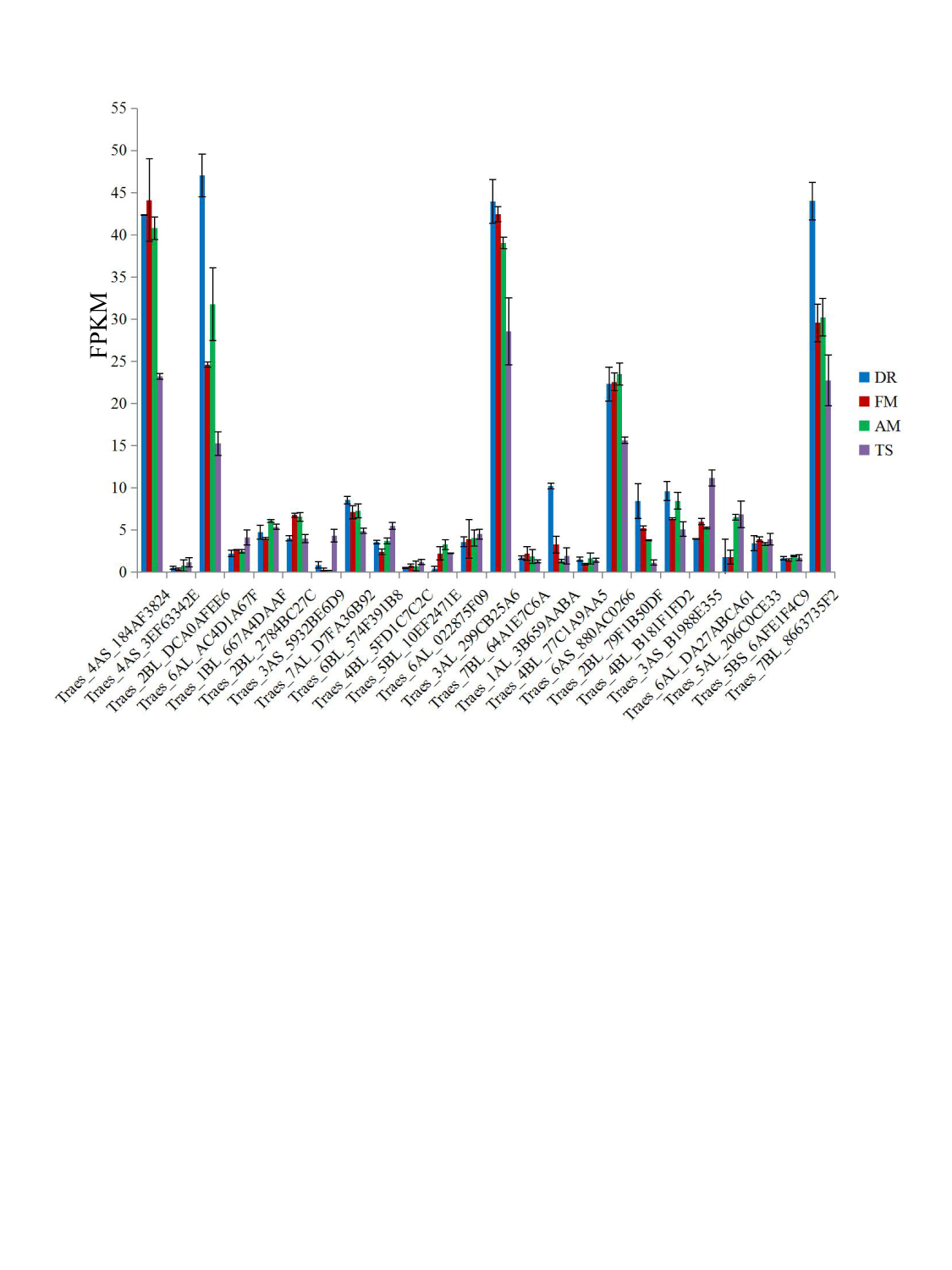

Supplement: FIGURE S4 — Expression patterns of spike development genes in the mutant L3090 with stop-gain mutations. [file Image_4.tif]

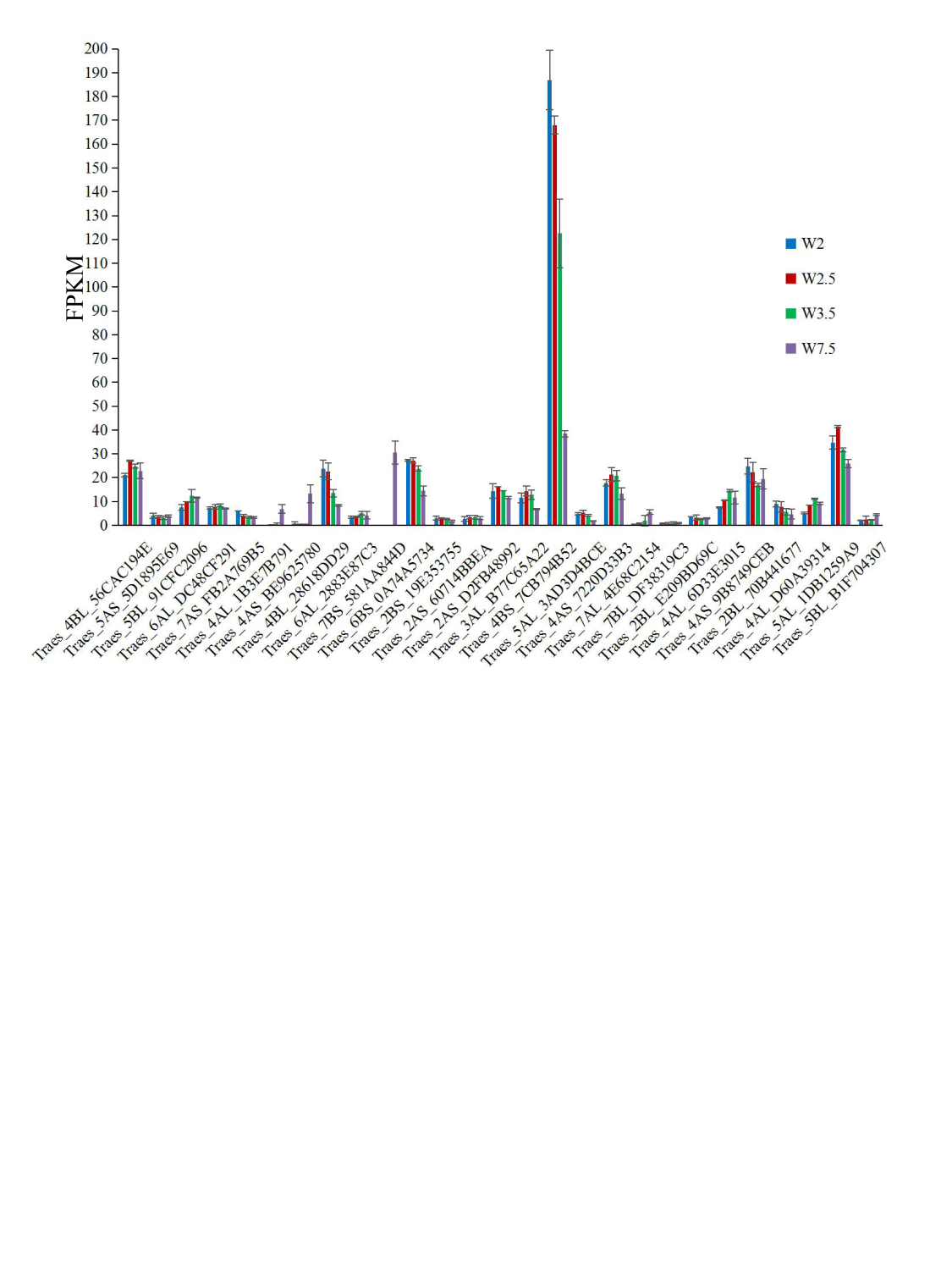

Supplement: FIGURE S5 — Expression patterns of spike development genes in the mutant L3090 with alternative splicing mutations. [file Image_5.tif]

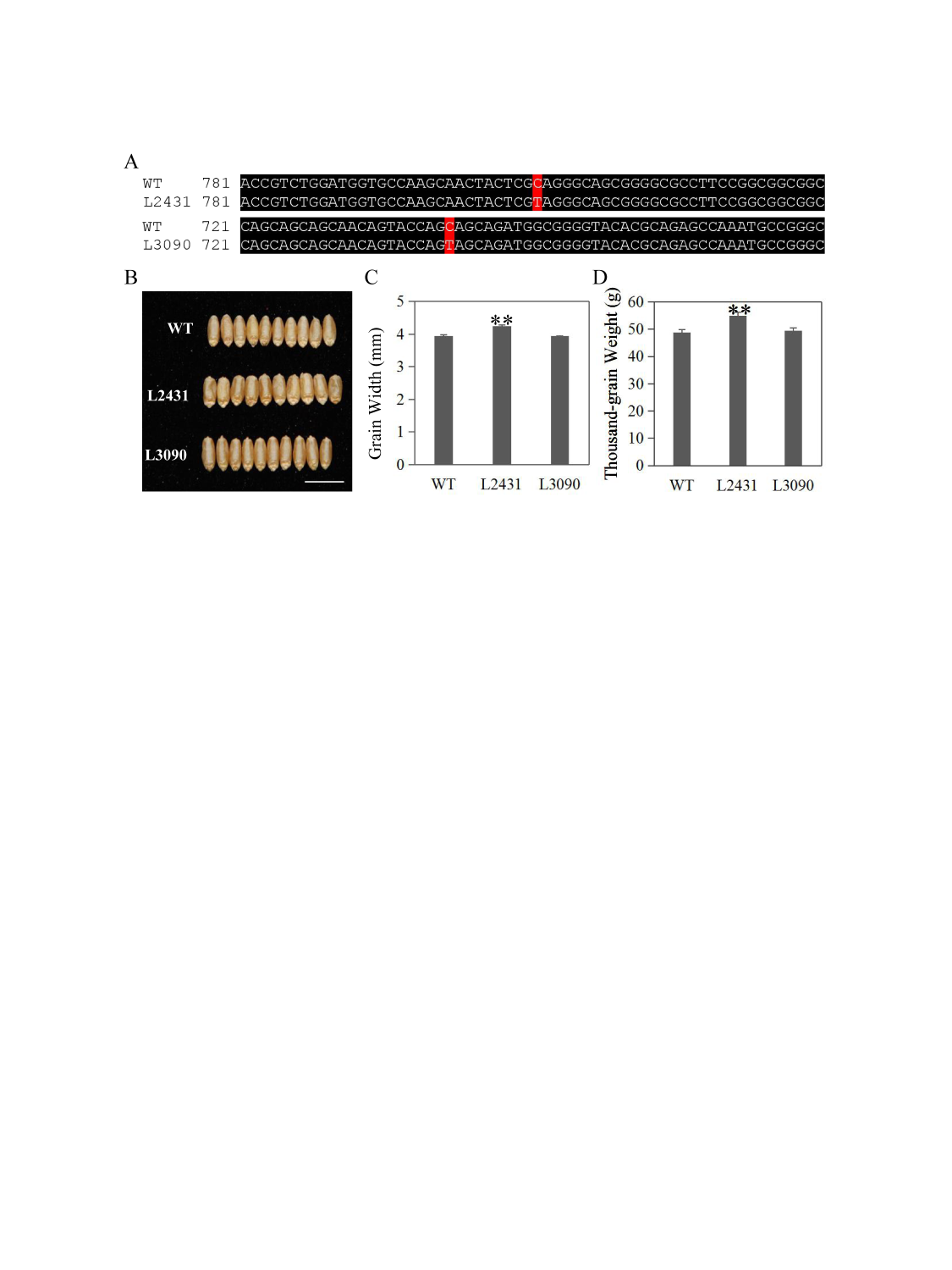

Supplement: FIGURE S6 — The effect of mutations of TaTCP9 on grain development. (A) Partial sequence alignment of TaTCP9 from wild type (WT) and two mutants L2431 and L3090, showing C811T in L2341 and C742T in L3090. (B) Grain width comparison between the mutants L2431 and L3090 and the wild type Kronos. (C,D) Statistic analysis of grain width (C) and thousand-grain weight (TGW) (D) in L2431 and L3090. Error bars indicate standard errors. A total of 23 spikes from six L2341 plants and 12 spikes from four L3090 plants were measured. Student’s t-test, ∗∗p < 0.01. Bar in panel B indicates 1 cm. [file Image_6.tif]
